# Effects of Fecal Microbiome Transfer in Adolescents With Obesity: The Gut Bugs Randomized Controlled Trial

**DOI:** 10.1001/jamanetworkopen.2020.30415

**Published:** 2020-12-21

**Authors:** Karen S. W. Leong, Thilini N. Jayasinghe, Brooke C. Wilson, José G. B. Derraik, Benjamin B. Albert, Valentina Chiavaroli, Darren M. Svirskis, Kathryn L. Beck, Cathryn A. Conlon, Yannan Jiang, William Schierding, Tommi Vatanen, David J. Holland, Justin M. O’Sullivan, Wayne S. Cutfield

**Affiliations:** 1 Liggins Institute, University of Auckland, Auckland, New Zealand; 2 A Better Start National Science Challenge, Auckland, New Zealand; 3 Children’s Hospital, Zhejiang University School of Medicine, Hangzhou, China; 4 Department of Women’s and Children’s Health, Uppsala University, Uppsala, Sweden; 5 Neonatal Intensive Care Unit, Pescara Public Hospital, Pescara, Italy; 6 School of Pharmacy, Faculty of Medical and Health Sciences, University of Auckland, Auckland, New Zealand; 7 School of Sport, Exercise and Nutrition, College of Health, Massey University, Auckland, New Zealand; 8 Department of Statistics, University of Auckland, Auckland, New Zealand; 9 Broad Institute of MIT and Harvard, Cambridge, Massachusetts; 10 Department of Infectious Diseases, Counties Manukau District Health Board, Auckland, New Zealand; 11 Maurice Wilkins Center, University of Auckland, New Zealand; 12 MRC Lifecourse Unit, University of Southampton, United Kingdom

## Abstract

**Importance:**

Treatment of pediatric obesity is challenging. Preclinical studies in mice indicated that weight and metabolism can be altered by gut microbiome manipulation.

**Objective:**

To assess efficacy of fecal microbiome transfer (FMT) to treat adolescent obesity and improve metabolism.

**Design, Setting, and Participants:**

This randomized, double-masked, placebo-controlled trial (October 2017-March 2019) with a 26-week follow-up was conducted among adolescents aged 14 to 18 years with a body mass index (BMI; calculated as weight in kilograms divided by height in meters squared) of 30 or more in Auckland, New Zealand. A total of 87 individuals took part—565 individuals responded to advertisements, 328 were ineligible, and 150 declined participation. Clinical data were analyzed from September 2019 to May 2020.

**Interventions:**

Single course of oral encapsulated fecal microbiome from 4 healthy lean donors of the same sex or saline placebo.

**Main Outcomes and Measures:**

Primary outcome was BMI standard deviation score at 6 weeks using intention-to-treat analysis. Secondary outcomes included body composition, cardiometabolic parameters, well-being, and gut microbiome composition.

**Results:**

Eighty-seven participants (59% female adolescents, mean [SD] age 17.2 [1.4] years) were randomized 1:1, in groups stratified by sex, to FMT (42 participants) or placebo (45 participants). There was no effect of FMT on BMI standard deviation score at 6 weeks (adjusted mean difference [aMD] −0.026; 95% CI −0.074, 0.022). Reductions in android-to-gynoid-fat ratio in the FMT vs placebo group were observed at 6, 12, and 26 weeks, with aMDs of −0.021 (95% CI, −0.041 to −0.001), −0.023 (95% CI, −0.043 to −0.003), and −0.029 (95% CI, −0.049 to −0.008), respectively. There were no observed effects on insulin sensitivity, liver function, lipid profile, inflammatory markers, blood pressure, total body fat percentage, gut health, and health-related quality of life. Gut microbiome profiling revealed a shift in community composition among the FMT group, maintained up to 12 weeks. In post-hoc exploratory analyses among participants with metabolic syndrome at baseline, FMT led to greater resolution of this condition (18 to 4) compared with placebo (13 to 10) by 26 weeks (adjusted odds ratio, 0.06; 95% CI, 0.01-0.45; *P* = .007). There were no serious adverse events recorded throughout the trial.

**Conclusions and Relevance:**

In this randomized clinical trial of adolescents with obesite, there was no effect of FMT on weight loss in adolescents with obesity, although a reduction in abdominal adiposity was observed. Post-hoc analyses indicated a resolution of undiagnosed metabolic syndrome with FMT among those with this condition. Further trials are needed to confirm these results and identify organisms and mechanisms responsible for mediating the observed benefits.

**Trial Registration:**

Australian New Zealand Clinical Trials Registry Identifier: ACTRN12615001351505

## Key Points

**Question:**

Will fecal microbiome transfer (FMT) lead to weight loss among adolescents with obesity?

**Findings:**

In this randomized clinical trial of 87 adolescents with obesity, FMT alone did not lead to weight loss at 6 weeks. FMT alone was associated with a reduction in android-to-gynoid-fat ratio sustained for at least 26 weeks, particularly in female adolescents; changes in the overall gut microbiome composition; and a resolution of metabolic syndrome by 26 weeks in participants who had this undiagnosed condition at baseline.

**Meaning:**

FMT alone is not an effective treatment for weight loss but may reduce visceral adiposity and improve health.

## Teaser

This randomized, double-masked, placebo-controlled clinical trial of 87 adolescents assesses the effect of fecal microbiome transfer on weight loss. Follow-up examination was conducted for the primary outcome at 6 weeks with intention-to-treat analyses, and was measured by body mass index standard deviation score.

## Introduction

Obesity has escalated into a global pandemic that affects an estimated one-tenth of the world’s adult population.^1^ As obesity tracks and increases through life,^2^ early intervention is important to reduce obesity-associated comorbidities and mortality.^3^

Most conventional treatments for obesity and obesity-associated diseases have been relatively unsuccessful.^4^ While lifestyle interventions and pharmacotherapy have led to weight reduction, the effects were small.^5^ Moreover, most drugs for treatment of obesity are unlicensed for pediatric use and have significant adverse effects.^5^ Similarly, although bariatric surgery results in weight reduction, severe postoperative complications such as bleeding and even death may occur.^6^

The gut microbiome plays a role in regulation of weight and metabolism by increasing energy extraction from food, altering energy expenditure, and modulating appetite and satiety, glucose homeostasis, and lipid metabolism in humans.^7,8^ Animal studies successfully altered body phenotypes by fecal microbiome transfer (FMT); germ-free mice that received microbiota from human donors with obesity developed obesity, whereas mice that received microbiota from human donors postbariatric surgery remained lean.^7,9,10,11^ This evidence highlights that FMT could be a treatment modality for human obesity.^4,10,11^

Evidence on the therapeutic benefits of FMT in obesity and metabolic syndrome is limited.^9^ Five studies examined the effect of FMT on insulin sensitivity and its safety among adults with obesity and metabolic syndrome.^12,13,14,15,16^ A temporary improvement in insulin sensitivity was demonstrated in 2 of these studies.^13,15^ Conversely, 2 other studies reported no effects on insulin sensitivity, but showed sustained gut microbiome changes after FMT.^12,16^ Notably, none of these studies examined the efficacy of FMT for weight loss as the primary outcome.^12,13,14,15,16^ Therefore, we assessed whether FMT using encapsulated fecal microbiome from lean donors would lead to weight loss and improve metabolism in adolescents with obesity.

## Methods

### Trial Ethics and Oversight

The Gut Bugs Trial was approved by the Northern A Health and Disability Ethics Committee (16/NTA/172). Adverse events were assessed at 24 and 48 hours and at 1, 3, 6, 12, and 26 weeks postintervention. Any reported adverse events were recorded and monitored. All participants and parents of those younger than age 16 years provided verbal and written informed consent. The trial was conducted from October 2017 to March 2019, and data were analyzed between September 2019 and May 2020. The trial protocol and statistical analysis plan are available in Supplement 1 and Supplement 2. The trial results were reported following the Consolidated Standards of Reporting Trials (CONSORT) reporting guideline.

### Trial Design and Randomization

This was a 2-arm randomized, double-masked, placebo-controlled trial, and the full trial protocol has been published.^4^ Participants were randomized in a 1:1 ratio to encapsulated fecal microbiome or saline placebo groups, stratified by sex. An independent researcher performed group allocation in 3 steps: (1) participant allocation to group A or B using the randomization sequence; (2) obtaining the capsule packs (independently labeled A or B by a technician); and (3) allocation of capsule packs to participants according to their unique code. Researchers and participants were masked to capsule contents during the trial. Blinding success was assessed using Bang’s Blinding Index (BBI).^4^

### Trial Participants and Intervention

Participants were ages 14 to 18 years, postpubertal, with obesity (body mass index [BMI; calculated as weight in kilograms divided by height in meters squared] 30 or greater), and without prediagnosed chronic diseases that could affect weight or metabolism. They were recruited through social media in Auckland, New Zealand. Clinical assessments were conducted at baseline and at 6, 12, and 26 weeks postintervention. Assessments included anthropometry, body composition, blood pressure, insulin sensitivity, metabolic markers, and gut microbiome profiling.^4^ BMI values were calculated and converted into standard deviation score (SDS) adjusted for age and sex using World Health Organization standards.^17^

Participants’ bowels were cleansed before the intervention using an oral 70-g bowel lavage solution (active ingredient macrogol 3350; Fresenius Kabi Australia Pty Ltd) the day before the intervention. Participants fasted overnight (≥8 h) before their clinic visit the next morning.

Fecal microbiome was extracted from stools of 8 healthy donors (4 men, 4 women) who were selected following a rigorous screening protocol.^4^ Fecal microbiome was double encapsulated using delayed-release hydroxypropyl methylcellulose capsules (Capsugel), which were designed to remain intact during passage through the stomach, delivering their contents to the intestine.^18,19,20^ Each participant received 7 capsules from each of the 4 same-sex donors.^4^ Participants therefore received 28 capsules, which equated to approximately 22 g (wet weight) of fecal material (approximately 14 mL of frozen microbial suspension or saline) over 2 consecutive days. After ingestion of capsules under direct supervision, participants remained fasted for another 2 hours. In addition to advice to not make changes to their usual diet, physical activity, and behavior, questionnaires were completed by the participants throughout the trial.^4^ At 6 weeks postintervention, participants completed a 3-day dietary record on food and fluids consumed; these records were entered into FoodWorks software version 9.0 (Xyris Software).^4^

### Outcome Measures

The primary outcome was BMI standard deviation score (BMI SDS) at 6 weeks postintervention. BMI SDS at 12 and 26 weeks postintervention were secondary outcomes. Other secondary outcomes included: total body fat percentage and android-to-gynoid-fat (A/G) ratio from whole-body dual-energy x-ray absorptiometry; blood pressure (including 24-hour ambulatory blood pressure monitoring); insulin sensitivity assessed by Matsuda index (from an oral glucose tolerance test) and HOMA-IR (Homeostatic Model Assessment of Insulin Resistance); metabolic markers (ie, lipid profile, liver function, and inflammatory markers) from plasma, and gut microbiome profiling from shotgun metagenomic sequencing.^4^ Participants also answered questionnaires on health-related quality of life (EPOCH Measure and Pediatric Quality of Life Inventory) and gut health (irritable bowel syndrome symptoms [Birmingham IBS] and bowel movements].^4^

### Statistical Analyses of Clinical Data

The primary outcome analysis was based on intention-to-treat with multiple imputations on missing data; the robustness of this analysis was subsequently assessed using the same linear regression without imputations. Detailed information on our multiple imputations procedures was provided in the published study protocol.^4^ For all continuous outcomes measured at 6, 12, and 26 weeks, linear mixed models with repeated measures were used to control for correlated data collected from the same participants. Binary outcomes were assessed using generalized linear models with a logit link. Models adjusted for the baseline outcome value and sex (stratification factor). Model-adjusted estimates and differences between groups were calculated with 95% confidence intervals. Planned subgroup analyses by sex were conducted on primary and secondary outcomes to evaluate the consistency of main treatment effects among male and female participants. Post-hoc exploratory analyses were also performed on the subgroup of participants who had undiagnosed metabolic syndrome at baseline.

Data were analyzed in SAS version 9.4 (SAS Institute) and SPSS version 25 (IBM Corp). Statistical tests were 2-sided at *P* < .05 without adjustments for multiple comparisons.

### Microbiome Analysis

Stool samples from participants were analyzed at baseline and at 6, 12, and 26 weeks postintervention. Processed stool capsules from donors were analyzed at each donation. Methods of DNA extraction have been described previously.^4^ The libraries for the metagenomic sequencing were prepared using DNA library prep kits for Illumina (NEB) and sequenced on an Illumina platform (Novogene) generating an average of 23 million reads per sample (150 bp paired-end reads). This was followed by quality control and removal of human reads using KneadData and taxonomic profiling with Metagenomic Phylogenic Analysis (MetaPhlAn2) version 2.7.7 (Segata Lab).^21^

The Shannon diversity index was used to estimate microbial diversity (ie, α diversity). Wilcoxon rank sum tests were used for 2-group comparisons, and Kruskal-Wallis rank sum tests were used for comparisons with more than 2 groups. Participant differences over time were assessed by Wilcoxon signed-rank tests. Community-level differences, based on Bray-Curtis dissimilarities, were assessed by permutational multivariate analysis of variance (PERMANOVA) using the adonis2 function in vegan R package version 3.6.1 (R Project for Statistical Computing). All PERMANOVA tests were adjusted for sex and ethnicity. Nominal *P* values from PERMANOVA were adjusted for multiple testing using the Benjamini-Hochberg procedure to obtain *q*-values, or the false discovery rate adjusted *P* value; results with *q* < 0.1 were considered statistically significant. When comparing donors and participants at baseline, multiple samples from each donor, corresponding to each donation batch, were averaged to generate a composite donor profile.

Associations between individual taxa and clinical variables were examined using generalized linear models as implemented in the Microbiome Multivariable with Linear Models (MaAsLin2) package.^22^ Relative abundance profiles were log-transformed, and taxa had to be present in at least 10% of samples to be included in analyses. The package’s default significance threshold of *q* < 0.25 was used to denote statistically significant differences in abundance of a given taxon. All models were adjusted for sex, and if multiple participant samples were included the participant identification number was added as a random effect.

## Results

**Figure 1.**
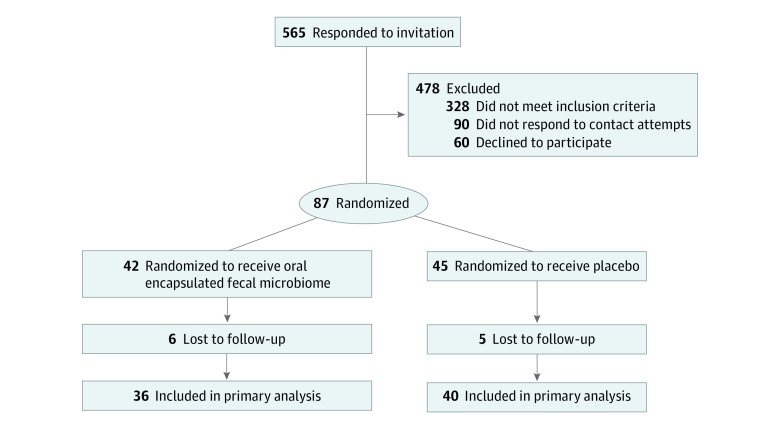
Flow Diagram of Participants Recruited and Randomized in the Gut Bugs Trial

From October 2017 to September 2018, 565 participants responded to advertisements, 328 were ineligible, 150 declined participation, and 87 were eligible (51 [58.6%] female adolescents, mean [SD] age 17.2 [1.4] years), and randomized into 2 groups; FMT (42 participants) or placebo (45 participants) (Figure 1). Participants were followed up over a period of 26 weeks postintervention, and all planned assessments were completed by March 2019. Baseline characteristics of the participants are shown in Table 1. No protocol violation was recorded on participants who provided the primary outcome data at 6 weeks. However, data from 2 participants at 26 weeks postintervention were excluded from analysis due to protocol violations (ie, started on ketogenic diet or probiotics). 95% of the participants’ data were available for the primary outcome (FMT, 39 participants; placebo, 44 participants), and 87% of the participants completed the trial at 26 weeks postintervention (Figure 1). A high mean (SD) daily consumption of sugar (87.7 [52.5] g) and saturated fat (32.6 [18.2] g) were reported with no differences observed between treatment groups.

**Table 1.** Baseline Characteristics of the Participants in the Gut Bugs Trial

Characteristics	Participants, No. (%)	
	FMT^a^	Placebo^a^
Total, No. (%)	42 (48)	45 (52)
Age, mean (SD), y	17.3 (1.5)	17.1 (1.4)
Female adolescents	25 (60)	26 (58)
Ethnicity		
New Zealand European	19 (45)	24 (53)
Māori	8 (19)	10 (22)
Pacific	10 (24)	10 (22)
Asian	5 (12)	1 (2)
Socioeconomic deprivation (IMD), quintile		
1 (least deprived)	2 (5)	4 (9)
2	8 (19)	12 (27)
3	12 (29)	10 (22)
4	7 (17)	8 (18)
5 (most deprived)	13 (31)	11 (24)
Anthropometry, mean (SD)		
Height, cm	172.7 (8.4)	172.5 (8.8)
Weight, kg	115.6 (23.4)	109.9 (16.3)
Waist circumference, cm	107 (13)	104 (10)
BMI	38.55 (5.92)	36.91 (4.61)
BMI SDS	3.46 (0.91)	3.21 (0.64)
Classes of obesity^b^		
I	12 (29)	17 (38)
II	16 (38)	17 (38)
III	14 (33)	11 (24)
Body composition, total body fat %, mean (SD)	47.4 (5.5)	47.6 (5.8)
Maternal characteristics		
Education, university degree or post–high school vocational qualification	23 (62)	32 (78)
BMI, mean (SD)	33.08 (7.87)	34.26 (7.76)
Paternal characteristics		
Education, university degree or post–high school vocational qualification	25 (68)	25 (60)
BMI, mean (SD)	31.15 (4.96)	32.78 (5.78)

Abbreviations: BMI, body mass index (calculated as weight in kilograms divided by height in meters squared); FMT, fecal microbiome transfer; IMD, Index of Multiple Deprivation; SDS, standard deviation score.

^a^ There were no observed differences between groups.

^b^ Obesity classes were defined as: Class I (BMI ≥30 and <35); Class II (BMI ≥35 and <40); and Class III (BMI ≥40).

### Clinical Outcomes

There was no observed effect of FMT on BMI SDS at 6 weeks postintervention based on intention-to-treat analysis with multiple imputations on missing data (adjusted mean difference [aMD], −0.026; 95% CI, −0.074 to 0.022; *P* = .291) or with the same linear regression without imputations (aMD, −0.025; 95% CI, −0.072 to 0.022; *P* = .295). In the FMT group, mean (SD) BMI SDS was 3.46 (0.91) at baseline, and 3.50 (0.97); in the placebo group, mean BMI SDS was 3.21 (0.64) at baseline and 3.29 (0.66) at 6 weeks. Neither was there an effect of FMT on BMI SDS at 12 or 26 weeks postintervention (Table 2). A reduction in A/G ratio was observed in the FMT group compared with placebo at 6, 12, and 26 weeks postintervention: with aMDs of −0.021 (95% CI, −0.041 to −.001; *P* = .042), −0.023 (95% CI, -0.043 to -0.003; *P* = .028), and −0.029 (95% CI, −0.049 to −0.008; *P* = .0069), respectively (Table 2). The improvement in A/G ratio was particularly marked among female participants, with a persistent reduction in A/G ratio at all time points (eTable 1 in Supplement 3).

**Table 2.** Secondary Analysis at 6, 12, and 26 Weeks Postintervention

Characteristic	Visit, wk	FMT		Placebo		aMD (95% CI)	*P* value
		Patients, No.	Mean (SD)	Patients, No.	Mean (SD)		
Anthropometry							
BMI SDS	0	42	3.46 (0.91)	45	3.21 (0.64)	NA	NA
	6^a^	39	3.50 (0.97)	44	3.29 (0.66)	−0.03 (−0.11 to 0.05)	.46
	12	38	3.46 (0.95)	41	3.29 (0.68)	−0.04 (−0.12 to 0.04)	.28
	26	37	3.51 (1.01)	41	3.30 (0.68)	−0.05 (−0.13 to 0.03)	.25
Waist circumference, cm	0	42	107.0 (13.0)	45	104.2 (10.2)	NA	NA
	6	39	107.1 (14.4)	44	105.4 (10.7)	−0.7 (−2.6 to 1.3)	.51
	12	38	106.6 (13.3)	41	105.4 (11.1)	−0.8 (−2.8 to 1.2)	.43
	26	37	106.0 (14.8)	41	104.5 (10.4)	−1.7 (−3.8 to 0.3)	.10
Body composition							
Total body fat, %	0	42	47.4 (5.5)	45	47.6 (5.8)	NA	NA
	6	39	47.5 (5.3)	44	47.7 (6.2)	−0.2 (−1.0 to 0.6)	.70
	12	38	47.8 (5.2)	41	47.7 (6.0)	−0.3 (−1.1 to 0.5)	.43
	26	37	47.3 (5.9)	41	47.5 (6.2)	−0.4 (−1.2 to 0.5)	.37
Total lean mass, kg	0	42	58.0 (12.5)	42	54.8 (8.9)	NA	NA
	6	39	57.9 (13.3)	39	54.9 (9.2)	0.3 (−0.7 to 1.3)	.58
	12	38	57.6 (12.6)	38	55.6 (9.4)	−0.1 (−1.1 to 0.9)	.87
	26	37	58.5 (12.7)	37	56.0 (9.7)	−0.5 (−1.5 to 0.6)	.39
A/G ratio	0	42	1.093 (0.116)	45	1.074 (0.084)	NA	NA
	6	39	1.081 (0.126)	44	1.088 (0.101)	−0.021 (−0.041 to −0.001)	.042
	12	38	1.083 (0.117)	41	1.095 (0.098)	−0.023 (−0.043 to −0.003)	.028
	26	37	1.075 (0.119)	41	1.088 (0.088)	−0.029 (−0.049 to −0.008)	.007
Glucose metabolism							
Fasting insulin, μIU/mL	0	42	33.8 (21.4)	45	30.7 (18.2)	NA	NA
	6	39	30.5 (16.0)	43	32.0 (19.6)	−3.9 (−9.9 to 2.1)	.21
	12	37	34.0 (18.4)	40	29.1 (16.5)	3.4 (−2.7 to 9.6)	.27
	26	36	37.3 (25.0)	40	31.7 (17.5)	3.1 (−3.0 to 9.3)	.31
Fasting glucose, mmol/L	0	42	5.36 (0.42)	45	5.33 (0.50)	NA	NA
	6	39	5.20 (0.48)	43	5.28 (0.56)	−0.11 (−0.32 to 0.10)	.29
	12	37	5.27 (0.48)	40	5.32 (0.55)	−0.11 (−0.32 to 0.10)	.30
	26	36	5.46 (0.40)	40	5.40 (0.48)	0.00 (−0.21 to 0.21)	.98
HOMA-IR	0	42	8.12 (5.34)	45	7.22 (4.01)	NA	NA
	6	39	7.14 (4.03)	43	7.60 (4.82)	−1.20 (−2.84 to 0.44)	.15
	12	37	8.10 (4.79)	40	6.93 (4.18)	0.55 (−1.13 to 2.23)	.52
	26	36	9.25 (6.70)	40	7.63 (4.29)	0.79 (−0.88 to 2.46)	.35
Matsuda index	0	42	1.58 (0.99)	44	1.75 (0.99)	NA	NA
	6	39	1.83 (1.18)	43	1.68 (0.83)	0.31 (−0.02 to 0.64)	.06
	12	37	1.67 (1.21)	40	1.92 (0.99)	−0.13 (−0.46 to 0.22)	.47
	26	36	1.57 (1.15)	40	1.62 (0.84)	0.06 (−0.28 to 0.40)	.74
HbA_1c_, mmol/mol	0	42	36.4 (4.4)	45	36.3 (4.5)	NA	NA
	6	39	36.1 (3.7)	43	36.1 (4.2)	0.1 (−1.1 to 1.3)	.83
	12	37	38.0 (2.7)	40	37.5 (2.8)	0.6 (−0.6 to 1.8)	.34
	26	35	38.8 (3.7)	39	39.5 (3.7)	−0.3 (−1.6 to 0.9)	.59
Clinic BP, mm Hg							
Mean SBP	0	42	119.6 (10.0)	45	116.9 (10.2)	NA	NA
	6	39	120.0 (11.7)	44	116.8 (11.7)	1.8 (−2.2 to 5.8)	.37
	12	38	120.2 (10.7)	41	118.0 (12.9)	1.2 (−2.9 to 5.3)	.56
	26	36	118.5 (8.2)	41	119.2 (9.3)	−2.6 (−6.8 to 1.6)	.22
Mean DBP	0	42	72.2 (8.0)	45	70.6 (10.4)	NA	NA
	6	39	71.2 (8.6)	44	69.3 (6.8)	1.2 (−1.8 to 4.3)	.44
	12	38	71.2 (9.3)	41	71.2 (7.8)	−0.4 (−3.6 to 2.7)	.78
	26	36	70.7 (7.9)	41	69.4 (7.8)	0.1 (−3.0 to 3.3)	.93
24hAMBP, mm Hg							
Mean SBP (SD)	0	42	115.5 (8.4)	45	115.0 (8.7)	NA	NA
	6	40	115.8 (9.2)	43	113.1 (8.1)	2.5 (0.3 to 4.7)	.02
Mean DBP (SD)	0	42	66.2 (4.7)	45	66.6 (5.2)	NA	NA
	6	40	66.1 (5.8)	43	65.1 (5.5)	1.3 (−0.4 to 3.1)	.14

Abbreviations: aMD, adjusted mean difference; A/G, android-to-gynoid fat; BMI SDS, body mass index standard deviation score; BP, blood pressure; DBP, diastolic blood pressure; FMT, fecal microbiome transfer; HbA_1c_, hemoglobin A_1c_; 24hAMBP, 24-hour ambulatory blood pressure monitoring; HOMA-IR, homeostatic model assessment of insulin resistance; NA, not applicable; SBP, systolic blood pressure.

To convert μIU/mL to pmol/L, multiply by 6.945; HbA_1c_ to proportion of total hemoglobin, multiply by 0.01 .

^a^ Further secondary analysis was conducted on this outcome at 6 weeks.

Metabolic parameters (insulin sensitivity by Matsuda index or HOMA-IR, liver function, lipid profile, and inflammatory markers), total body fat percentage, and clinic blood pressure were not affected by FMT at 6, 12, and 26 weeks postintervention (Table 2 and Table 3; eTable 2 in Supplement 3). At 6 weeks, the systolic blood pressure from 24-hour ambulatory blood pressure monitoring was slightly lower (aMD, 2.5 mm Hg) in the placebo group (95% CI, 0.3-4.7; *P* = .024) (Table 2). There were no differences between FMT or placebo group for gut health and health-related quality of life.

**Table 3.** Secondary Binary Outcomes at 6, 12, and 26 Weeks Postintervention

	Visit, wk	FMT patients		Placebo patients		aOR (95% CI)	*P* value
		No.	No. (%)	No.	No. (%)		
Elevated clinic BP^a^	0	42	10 (24)	45	8 (18)		
	6	39	9 (23)	44	10 (23)	0.85 (0.27-2.65)	.77
	12	38	8 (21)	41	7 (17)	1.19 (0.35-4.11)	.78
	26	36	3 (8)	41	7 (17)	0.20 (0.03-1.14)	.07
Abnormal glycaemia^a^	0	41	22 (54)	44	23 (52)		
	6	38	20 (53)	43	19 (44)	1.32 (0.48-3.66)	.59
	12	37	21 (57)	40	24 (60)	0.84 (0.30-2.39)	.75
	26	35	24 (69)	40	28 (70)	0.92 (0.31-2.78)	.88
Abnormal liver function^a^	0	42	14 (33)	45	8 (18)		
	6	39	14 (36)	43	10 (23)	1.78 (0.65-4.89)	.26
	12	37	12 (32)	40	8 (20)	1.83 (0.63-5.38)	.27
	26	36	15 (42)	40	10 (25)	2.02 (0.73-5.57)	.17
Dyslipidaemia^a^	0	41	30 (73)	45	34 (76)		
	6	37	28 (76)	43	29 (67)	1.60 (0.56-4.53)	.38
	12	34	22 (65)	40	31 (78)	0.59 (0.20-1.70)	.32
	26	35	23 (66)	40	33 (83)	0.49 (0.16-1.50)	.21
Metabolic syndrome^a^^,^^b^	0	41	18 (44)	45	13 (29)		
	6	37	18 (49)	43	16 (37)	1.25 (0.43-3.65)	.68
	12	34	10 (29)	40	13 (33)	0.68 (0.21-2.21)	.52
	26	35	9 (26)	40	13 (33)	0.50 (0.15-1.66)	.26

Abbreviations: aOR, adjusted odds ratio; BP; blood pressure; FMT, fecal microbiome transfer.

^a^ All outcomes defined in eTable 6 in Supplement 3.

^b^ There was a larger reduction in prevalence of metabolic syndrome with FMT in those who had metabolic syndrome at the start of the study (see eTable 4 in Supplement 3). In the overall analyses, participants from both groups developed metabolic syndrome during the course of the study.

### Post-Hoc Exploratory Analyses

We carried out a post-hoc exploratory analyses of the subgroup of adolescents who had undiagnosed metabolic syndrome at baseline. While there was a marked difference in BMI SDS at baseline (BMI SDS greater in FMT group: aMD, 0.91; 95% CI, 0.34-1.49; *P* < .001), such differences disappeared by 6 weeks postintervention (eTable 3 in Supplement 3). FMT led to improvements in insulin sensitivity and glucose metabolism at 6 weeks postintervention, with participants showing a 34% improvement in HOMA-IR (aMD, 0.66; 95% CI, 0.46-0.93; *P* = .018), a 29% reduction in fasting insulin (aMD, 0.71; 95% CI, 0.51-0.99; *P* = .042), and a 7% reduction in fasting glucose (aMD, −0.38; 95% CI, −0.73 to −0.03; *P* = .033) (eTable 3 in Supplement 3) which did not persist at subsequent visits (eTable 3 in Supplement 3). A majority of participants in the FMT-treated group had resolution of metabolic syndrome (78%, from 18 to 4 participants) by 26 weeks postintervention, with an adjusted odds ratio (aOR) of 0.06 (95% CI, 0.01-0.45; *P* = .0074) (eTable 4 in Supplement 3).

### Gut Microbiome Differences Between Donors and Participants at Baseline

**Figure 2.**
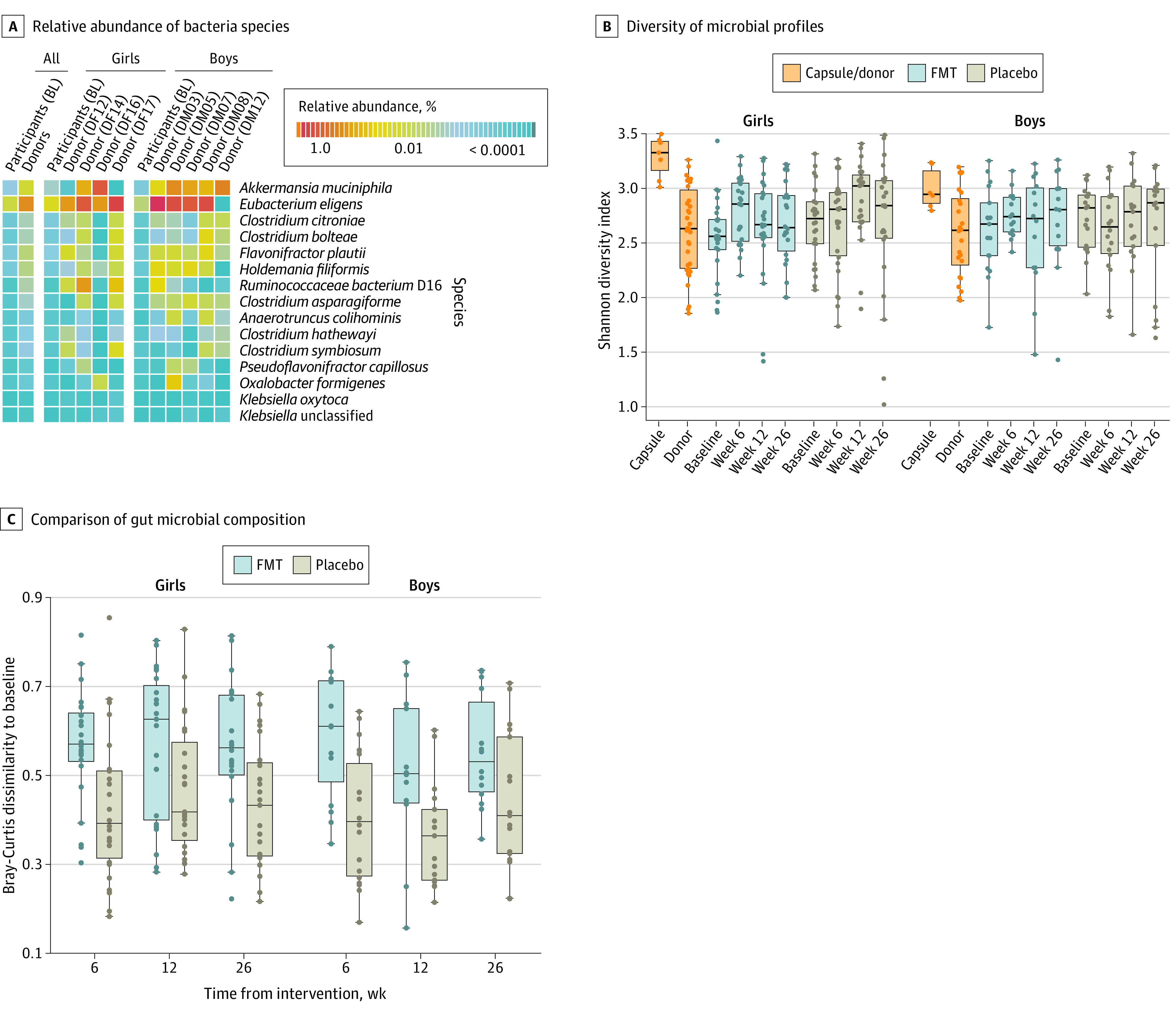
Gut Microbiome Assessment of Donors and Participants Boxes indicate interquartile ranges (IQR), and whiskers the range of the data (expanding up to 1.5 × IQR). Each dot indicates an individual’s fecal microbiome sample, except for capsule dots, which are the combination of all 4 contributing donor microbiome samples per batch. FMT indicates fecal microbiome transfer.

Profiling of the gut microbiome using metagenomic sequencing revealed differences in community composition between donors and participants at baseline. At the phylum level, we did not observe any difference in Firmicutes to Bacteroidetes ratio (median [IQR] ratio: donors, 1.90 [2.52] vs recipient, 2.16 [6.14]; *P* = .65). At the species level, we observed differences in the abundance of a number of taxa. Donors had an increased abundance of *Akkermansia muciniphila* (a species commonly associated with obesity protection^24^) (*q* < 0.25) (Figure 2).

Despite the difference in overall community structure, microbial diversity was similar between individual donors and participants at baseline (Figure 2). However, FMT treatment used capsules from all 4 donors at once. Analysis confirmed that the microbial profiles of the combined capsule sets were more diverse than those of the individual donor and participant samples at baseline (Figure 2).

### Shifts in Participant’s Microbiota Composition Post-Intervention

FMT intervention affected the overall gut community composition at weeks 6 and 12 post-intervention (q <0.10) (eTable 5 in Supplement 3). The impact of treatment was manifest in the significantly greater dissimilarity between the postintervention and baseline gut microbial composition observed in the FMT-treated, but not placebo, participants (Figure 2). A significant increase in microbial diversity was observed at 6 weeks post-FMT in female participants but this difference was not sustained at the later time points (Figure 2). Among male participants, we did not observe an increase in microbial diversity at any time points post-FMT.

Within the FMT group, improvements in A/G ratio were associated with a reduction in the relative abundance of *E coli*, and an increase in *Faecalibacterium prausnitzii, Bacteroides ovatus, Bacteroidales bacterium ph8, Alistipes onderdonkii, Alistipes finegoldii,* and *Alistipes shahii* (q < 0.25). Changes in A/G ratio were weakly correlated with post-FMT microbial diversity and improvements were associated with higher diversity, although not significant (Spearman’s rank correlation, ρ = −0.17, *P* = .072). Additionally, there were no differences in the gut microbial diversity or composition at 26 weeks postintervention among those individuals who had a FMT and resolved or maintained their metabolic syndrome status.

### Adverse Events

There were no serious adverse events recorded throughout the trial. Although minor adverse events occurred, these events were rare and were not related to treatment. The most frequently reported minor adverse event was loose stools with 53 (10.0%) positive responses out of 529 recorded responses. This was followed by 46 (8.7%) for changes in frequency of bowel movements, 39 (7.4%) for abdominal pain, 22 (4.2%) for nausea/vomiting, 20 (3.8%) for excessive and/or malodorous burps, 9 (1.7%) for fever, and 2 (0.4%) for bloody stools. Notably, no participants reported any discomfort or difficulty ingesting the required number of treatment capsules.

### Masking

There was successful masking of the FMT group (BBI, −0.05; 95% CI, −0.28 to 0.17); only 23% correctly identified their treatment, while 28% believed they were taking placebo, and 49% indicated that they did not know the treatment they were on. Similarly, the majority of participants in the placebo group did not know the treatment they were on (BBI, 0.27; 95% CI, 0.11 to 0.43).

## Discussion

We showed that FMT from lean donors did not influence body weight in adolescents with obesity. A reduction in A/G ratio was observed post-FMT at 6 weeks and sustained for at least 26 weeks particularly among female participants. We also demonstrated a sustained change in gut microbiome composition of participants for up to 12 weeks following a single FMT treatment. This change was accompanied by increased gut microbiome diversity 6 weeks post-FMT among female participants. In addition, post-hoc analyses among those with metabolic syndrome at baseline showed transient improvements in glucose metabolism and insulin sensitivity at 6 weeks, and resolution of metabolic syndrome in most FMT-treated participants by 26 weeks.

Our negative findings on weight loss were consistent with previous clinical trials of FMT in adults.^12,13,14,15,16^ However, those trials examined body weight as a secondary outcome, and were not powered to detect effects on body weight.^9,12,13,14,15,16^ Our finding of a small reduction in A/G ratio, and therefore a likely reduction in visceral adiposity is a new observation that has not been reported in previous studies.^12,13,14,15,16^ This finding of improved A/G ratio with FMT corresponded to an approximately 2% to 3% reduction in the proportion of abdominal fat, and was particularly marked among female participants. A greater A/G ratio has been associated with insulin resistance, adverse lipid profiles,^25^ and predictive of cardiometabolic health.^26^ While the long-term significance of this reduction remains to be determined, the observed impact of the FMT raises promising new avenues to investigate the complex relationships between lipid partitioning and metabolic health.

Two studies of adult males with metabolic syndrome^13,15^ demonstrated that, following a single FMT treatment, there were changes in gut microbiome composition and/or diversity at 6 weeks and improvement in insulin sensitivity at 6 weeks, which were not sustained at 18 weeks. In our study, FMT led to alterations in gut microbiome at 6 weeks that were sustained until 12 weeks, but which were not associated with changes in insulin sensitivity. In a 2019 study,^27^ a more targeted approach was used to trial the efficacy of *Akkermansia muciniphila* supplementation in adults who were overweight, reporting an improvement in insulin sensitivity assessed by an indirect measurement (ie, HOMA-IR). In our post-hoc subgroup analyses of participants with the metabolic syndrome at baseline, FMT led to a transient improvement in glucose metabolism at 6 weeks and a reduction in the rate of metabolic syndrome by the end of the follow-up period (ie, 26 weeks). Collectively, our results and the findings from these studies^13,15,27^ suggest that FMT or targeted microbial treatment may be effective in alleviating features of metabolic syndrome, but weekly treatments every 6 weeks may be required for those with this condition.

There was a prominent and sustained change in the gut microbiome composition of the FMT-treated group at both community and individual taxa levels. The improvements in A/G ratio in the FMT group were associated with a reduction in relative abundance of *E coli*, and an increase in *F prausnitzii* and *Alistipes* spp. All of these organisms have been previously linked to BMI.^28,29^ Increased abundance of *E coli* has been associated with higher BMI and increased ferritin levels (a protein linked to chronic low-grade inflammation in obesity).^28^ *F prausnitzii* has been associated with anti-inflammatory effects and improved gut barrier function in lean individuals.^29^ Additionally, the abundance of *Alistipes* spp changed in individuals who successfully maintained weight loss after a weight loss interventional trial.^29^ Therefore, we speculate that the changes observed in the levels of these organisms likely contributed to the improvements in A/G ratio.

We employed a noninvasive method of FMT delivery using encapsulated fecal microbiome, which prevented adverse events associated with the physical delivery of FMT using nasojejunal tube or colonoscopy.^9^ No major adverse events were observed with FMT and although minor adverse events occurred, these events were rare. Notably, with the successful masking of the FMT group and no reported discomfort from any participant after ingesting the full dose of FMT capsules, we demonstrated that capsules were an effective, safe, and acceptable method of FMT delivery. In contrast to other studies whereby participants received FMT from different donors, we were able to standardize treatment by using repeated donations from the same donors throughout the trial and thus reducing potential confounders because of donor-specific effects.^9,12,13,16^

### Limitations

This study had several limitations. First, a limitation of our study was that diet and physical activity were not tightly regulated. In mice studies where diet was carefully regulated, a reduction in weight was associated with FMT treatment and the beneficial effect of FMT can be negated by a poor diet.^10^ Our trial participants reported a high consumption of saturated fat and sugar consistent with previous observations on dietary intake of adolescents with obesity in New Zealand.^30^ Mitigating this limitation would have required continuous residential supervision of all food intake and physical activity. In future trials, an initial period of structured dietary counselling preintervention, supervised dietary intake, and regular monitoring and follow-up with trained dietitians postintervention should be considered. Second, in our post-hoc analyses on adolescents with the metabolic syndrome, participants in the 2 groups had discordant BMI SDS at baseline, but this difference disappeared by 6 weeks; it is unclear whether this change was associated with FMT or just regression to the mean, which needs to be taken into account when interpreting these findings.

## Conclusions

This randomized clinical trial demonstrated that FMT alone did not reduce BMI SDS in adolescents with obesity, but led to a reduction in abdominal adiposity. Further, post-hoc subgroup analyses suggested that FMT was associated with transient metabolic improvements and resolution of metabolic syndrome in many participants with this condition preintervention; however, these findings require confirmation from other trials focusing specifically on participants with the metabolic syndrome. We believe that FMT can be a feasible future treatment for obesity and/or metabolic diseases, but it is likely targeted microbial therapy with a defined, cultured microbial mixture would be more socially acceptable and safe. Future trials should therefore focus on identifying the organisms and mechanisms that were responsible for mediating the observed benefits in the presence of dietary restriction.

## Notes

Supplement 1. **Trial Protocol**

Supplement 2. **Statistical Analysis Plan**

Supplement 3. **eTable 1.** Android-to-Gynoid-Fat (A/G) Ratio at 6, 12, and 26 Weeks Postintervention Among Female and Male Adolescents **eTable 2.** Secondary Outcomes at 6, 12, and 26 Weeks Postintervention **eTable 3.** BMI SDS and Glucose Metabolism of Participants with Metabolic Syndrome at Baseline **eTable 4.** Changes in Prevalence of Metabolic Syndrome Among Participants with Metabolic Syndrome at Baseline **eTable 5.** Variance of Species Taxonomic Profiles (Bray-Curtis Dissimilarity) Explained by Each Covariate in Cross-sectional PERMANOVA **eTable 6.** Variance of Species Taxonomic Profiles (Bray-Curtis dissimilarity) Explained by Each Covariate in PERMANOVA for Donors and Recipients at Baseline **eTable 7.** Definitions of Abnormal Outcomes **eReferences.**

Supplement 4. **Data Sharing Statement**
