# Supplementary material for: Effects of Fecal Microbiome Transfer in Adolescents With Obesity: The Gut Bugs Randomized Controlled Trial
Source: JAMA Netw Open. 2020 Dec 21;3(12):e2030415. doi: 10.1001/jamanetworkopen.2020.30415 (PMC7753902; doi:10.1001/jamanetworkopen.2020.30415)
Supplement: Supplement 1. — Trial Protocol [file jamanetwopen-e2030415-s001.pdf]

Protocol for the Gut Bugs Trial: a randomised double-blind placebo-controlled trial of gut microbiome transfer for the treatment of obesity in adolescents

Karen S W Leong[1,2], Thilini N Jayasinghe[1], José G B Derraik[1,2,3], Benjamin B Albert[1,2], Valentina Chiavaroli[1], Darren M Svirskis[4], Kathryn L Beck[5], Cathryn A Conlon[5], Yannan Jiang[6], William Schierding[1], Tommi Vatanen[1,7], David J Holland[8], Justin M O'Sullivan[1,2†], Wayne S Cutfield[1,2†]

[1] Liggins Institute, University of Auckland, Auckland, New Zealand.
[2] A Better Start – National Science Challenge, University of Auckland, Auckland, New Zealand.
[3] Department of Women's and Children's Health, Uppsala University, Uppsala, Sweden.
[4] School of Pharmacy, Faculty of Medical and Health Sciences, University of Auckland, Auckland, New Zealand.
[5] School of Sport Exercise and Nutrition, College of Health, Massey University, Auckland, New Zealand.
[6] Department of Statistics, University of Auckland, Auckland, New Zealand.
[7] Broad Institute of MIT and Harvard, Cambridge, Massachusetts, USA.
[8] Department of Infectious Diseases, Counties Manukau District Health Board, Auckland, New Zealand.

[†]JM O'Sullivan and WS Cutfield contributed equally to the study.

Corresponding author: Professor Wayne Cutfield, Liggins Institute, University of Auckland, Private Bag 92019, Auckland, New Zealand; e-mail w.cutfield@auckland.ac.nz; ph +64.9.923.4476; fax +64.9.373.8763.

Competing interests: The authors have no financial conflicts of interest to disclose that may be relevant to this work. The funders had no role in study design, decision to publish, or preparation of this manuscript.

Word count: 5,405

This study protocol has been published in BMJ Open and is available at https://bmjopen.bmj.com/content/9/4/e026174.long

ABSTRACT

Introduction: Animal studies showed that germ-free mice inoculated with normal mouse gut bacteria developed obesity, insulin resistance, and higher triglyceride levels, despite similar food intake. In humans, an association has been found between obesity and gut microbiome dysbiosis. However, gut microbiome transfer has not been evaluated for the treatment of human obesity. We will examine the effectiveness of gut microbiome transfer using encapsulated material for the treatment of obesity in adolescents.

Methods and analysis: A two-arm, double-blind, placebo-controlled, randomised clinical trial of a single course of gut microbiome transfer will be conducted in 80 obese (BMI ≥30 kg/m^2) adolescents (males and females, aged 14–18 years) in Auckland, New Zealand. Healthy lean donors (males and females, aged 18–28 years) will provide fresh stool samples from which bacteria will be isolated and double encapsulated. Participants (recipients) will be randomised at 1:1 to control (placebo) or treatment (gut microbiome transfer), stratified by sex. Recipients will receive 28 capsules over two consecutive mornings (~14 ml of frozen microbial suspension or saline). Clinical assessments will be performed at baseline, 6, 12, and 26 weeks, and will include: anthropometry, blood pressure, fasting metabolic markers, dietary intake, physical activity levels, and health-related quality of life. Insulin sensitivity (Matsuda index), gut microbiota population structure characterized by 16S rRNA amplicon sequencing, and body composition (DXA) will be assessed at baseline, 6, 12, and 26 weeks. 24-hour ambulatory blood pressure monitoring will be performed at baseline and at 6 weeks. The primary outcome is BMI standard deviation scores (SDS) at 6 weeks, with BMI SDS at 12 and 26 weeks as secondary outcomes. Other secondary outcomes include insulin sensitivity, adiposity (total body fat percentage), and gut microbial composition at 6, 12, and 26 weeks. Statistical analysis will be performed on the principle of intention to treat.

Ethics and dissemination: Ethics approval was provided by the Northern A Health and Disability Ethics Committee (HDEC) (Ministry of Health, New Zealand; 16/NTA/172). The trial results will be published in peer-reviewed journals and presented at international conferences.

Trial registration number: ACTRN12615001351505

Strengths of this study

- This is the largest registered randomised clinical trial of gut microbiome transfer for obesity or insulin resistance in children or adults.
- The double-blind, placebo-controlled design, use of capsules as a non-invasive method of delivery, and characterisation of bacterial diversity and viability in donor stools are main strengths of this randomised clinical trial.
- Conducting a 6-month follow-up after a single treatment with gut microbiome will allow identification of a possible lag between treatment and change in BMI.
- This study is adequately powered to show a meaningful reduction in BMI SDS in the treated group.

Limitations of this study

Our study will focus on obese adolescents, so that the findings may not be readily extrapolated to individuals with lesser degrees of adiposity or to older adults.

INTRODUCTION

There is an increasing prevalence of obesity amongst children and adolescents[1]. Obesity tracks and amplifies through life[2 3], and childhood obesity is associated with even greater severity of obesity and related co-morbidities in adulthood[2 4]. An elevated body mass index (BMI) in adolescence is associated with an increased all-cause mortality in adult life, and it is more predictive of later mortality than an elevated adult BMI[5 6].

New paradigms on the causes of obesity incorporate a pivotal role for the gut microbiota (*i.e.* the microbial community present in the gastrointestinal tract). In recent years, assessments of the gut microbiome (all of the genes inside these gut microbiota cells) have identified reduced diversity of bacterial taxa as having an important effect on the development of obesity, insulin resistance, and diabetes mellitus[7 8]. The concept that the gut microbiome influences host metabolism and adiposity was introduced through gut microbiome transfer experiments in gnotobiotic (*i.e.* germ-free) mice[9 10]. These gnotobiotic mice have sterile colons and 40% less body fat than conventional mice, despite a food intake that is 29% higher[9]. When germ-free mice were inoculated with normal mouse gut bacteria, they developed obesity, insulin resistance, and increased triglycerides levels, while on the same food intake[11]. Further experiments showed that the gut microbiome modulates both sides of the energy balance equation by: (i) increasing energy yield from the diet stored as triglycerides; and (ii) altering energy expenditure via fatty acid oxidation[10 12 13]. These effects occur either directly within the bowel, or indirectly through the effects of bacterial products that enter the circulation. Current literature

indicates that changes to the gut microbiome and their respective products within the host circulation (*e.g.* lipopolysaccharides and short-chain fatty acids) can alter host responses, modulate insulin resistance, adiposity, and atherosclerosis and have an effect on the development of non-alcoholic fatty liver disease[14 15].

Gut microbiome transfer is now regularly used to treat recurrent or refractory *Clostridium difficile* colitis, which is associated with considerable morbidity and a reported 38% mortality[16]. Other treatment regimens for this disorder have relied on repeated courses of vancomycin, typically with low cure rates (~31%)[17]. By contrast, a single naso-duodenal infusion of a 'healthy' gut microbiome in elderly patients with chronic *C. difficile* colitis led to cure in 81% of subjects[17]. This [17] and other studies[18-20] have demonstrated that gut microbiome transfer is a viable treatment option for recurrent or refractory *C. difficile* colitis, without any noticeable side effects. Studies have confirmed that 6 weeks after gut microbiome transfer, the recipient's gut microbiome population structure resembles that of the donor[21].

Gut microbiome transfer is a possible treatment for obesity and metabolic syndrome[22]. To date, investigation of the therapeutic benefit of gut microbiome transfer in adult metabolic disease (obesity and metabolic syndrome) has been limited[20]. Vrieze et al. performed a short-term gut microbiome transfer study in 9 treated and 9 control middle-aged adults with metabolic syndrome[20]. Six weeks after gut microbiome transfer via naso-duodenal tube, treated recipients had an impressive 75% improvement in insulin sensitivity. Kootte et al. reported similar results at 6 weeks among 38 obese males (median age 56 years), but the improvements in both insulin sensitivity and gut microbiota composition reverted back to baseline at 18 weeks [23]. Conversely, our group (unpublished data) demonstrated that gut microbiome composition in recipients changed after gut microbiome transfer to mimic the lean donor's gut microbiome, and that this effect was sustained 26 weeks after treatment. This indirectly indicates that it is possible to change the gut microbiome, using a healthy donor, with possible concurrent health benefits.

Selection of donors is critical for successful gut microbiome transfer. The adverse effect of an inappropriate donor was illustrated by a patient with chronic *C. difficile* colitis, who developed new-onset obesity following gut microbiome transfer from a healthy but overweight donor[24]. Notably, a similar result was observed when the microbiome from an obese human was transferred into a lean mouse[25].

Gut microbiome transfer is not considered a probiotic treatment[26]. Although gut microbiome transfer and probiotics involve the administration of live bacteria, this is where the similarities end. Probiotics are one of several defined live bacterial strains (*e.g. Bifidobacterium adolescentis*, *Lactobacillus acidophilus*, and *Lactobacillus casei*) that have been previously isolated and characterised[26]. The

rationale for this treatment is that these supplemental bacteria and products have been shown to confer general health benefits. Conversely, gut microbiome transfer consists of transferring the entire microbiome from a healthy donor to a recipient, in order to establish a healthier microbial community and ameliorate the undesirable underlying condition. Meta-analyses of randomised control studies of the effects of probiotics (*e.g. Lactobacillus* spp. and fermented milk-based probiotic treatments) on weight loss are conflicting[27 28]. There are currently no published studies of gut microbiome transfer for the treatment of human obesity. However, a study has shown that germ-free mice lose weight following gut microbiome transfer from mice who had gastric bypass surgery and exhibited rapid weight loss[29]. In addition, meta-analyses of the effectiveness of microbial transfers in the treatment of *C. difficile*[30] have demonstrated that gut microbiome transfer is efficacious and safe for inflammatory bowel disease (pooled cure rate 36%; 95% CI 17–60%)[31] and *C. difficile* (pooled cure rate 89%; 95% CI 84–93%)[30]. As such, gut microbiome transfer holds significant promise as a treatment for the rapid and concerted modification of an unhealthy gut microbiome, which we hypothesise will lead to weight loss in obese humans.

This clinical trial will assess whether gut microbiome transfer using encapsulated material is an effective treatment for obesity in adolescents.

METHODS AND ANALYSIS

Study design

A two-arm, double-blind, placebo-controlled, randomised clinical trial with obese adolescents randomly assigned to either treatment (encapsulated gut microbiome) or placebo (encapsulated saline solution), stratified by sex. Eligible participants will be followed for 26 weeks post randomisation (Figure 1). This trial protocol is reported as per the SPIRIT guidelines [32].

Recruitment and eligibility criteria

Donors

We will recruit 8 donors (4 males and 4 females), as recipients will only receive gut microbiome from donors of the same sex. This is to enhance microbial variability and standardise the treatment via gut microbiome transfer. Treatment with gut microbiome from donors of the same sex will be done as there may be potentially sex-specific differences in the effect of gut microbiome on weight and metabolism as described by Markle et al. [33]. Donors will be selected based on strict inclusion criteria (Table 1). Eligible donors will be identified by word of mouth, the internal email system at the University of

Auckland, and social media networks. Potential donors will be given a detailed information sheet about the study that includes a consent form.

To eliminate the risks of transmission of infectious diseases we will use screening procedures equivalent to those used for blood donation in New Zealand[34], and also screen donors for potential faecal pathogens or multidrug-resistant organisms. As part of this regimen, all potential donors will undergo extensive testing for human pathogens, antigens, and antibodies (that indicate exposure to hepatitis A, B, or C viruses, and human immunodeficiency virus), syphilis, *C. difficile*, *Helicobacter pylori*, other bacterial and viral pathogens, multidrug-resistant organisms, as well as intestinal parasites. We will supplement these microbiological tests with characterisation of the gut microbiome through analysis of the metagenome and metatranscriptome[35]. In addition, we will conduct an interview to gather information about behaviours or activities that may exclude them from the trial (Table 1).

Given evidence that irritable bowel syndrome (IBS) may be related to the gut microbiome, it is important to exclude potential donors who may have IBS. The Rome criteria are an accepted clinical tool to identify individuals with IBS, but they are relatively insensitive so that strict adherence to those criteria would potentially allow for individuals with mild IBS to donate[36]. Therefore, we will screen for IBS using a conservative modification of the Rome criteria, where we define a positive screen as having 3 or more episodes of abdominal pain per month as described in part I of the criteria, as well as an additional symptom as defined in part II[37].

Each donor is expected to produce a wet stool sample weighing 100-150 g. Our preliminary laboratory data indicate that an average stool sample from a donor will generate sufficient gut microbiome material for two same-sex recipients. Stool samples will be collected and immediately processed for encapsulation. Capsules from each sample will be individually coded, so that each recipient will receive an equal number of capsules (n=7) from each of the four same sex donors.

Participants (recipients)

We will recruit 80 obese adolescents as per inclusion and exclusion criteria described in Table 2. Eligible recipients will be recruited via social media, word of mouth, and paediatric endocrinology clinics in Auckland. Potential recipients and caregivers will be given a detailed information sheet about the study that includes a consent form. Consent will be obtained from recipients if they are aged ≥16 years and from their parents if aged <16 years. Younger recipients will also be asked to sign an assent form. All consent and/or assent will be obtained by the researchers prior to the recipient's participation in the trial. All potential and enrolled recipients' personal information are recorded and kept in a secure folder and only accessible to the researchers, in order to protect their confidentiality.

Specimen collection

The donor gut microbiome will be double encapsulated and administered to recipients by the oral route, which delivers bacteria to the proximal bowel. Thus, we will not require the use of invasive techniques (*i.e.* naso-duodenal tube) for gut microbiome transfer. Instead, gut microbiome transfer will be performed as per recent studies[18 19], which demonstrated that an encapsulated microbiome was a viable treatment option for recurrent or refractory *C. difficile* colitis, without noticeable side effects.

We have validated methods for gut microbiome isolation, preparation, and double encapsulation as detailed by Youngster et al.[18]. Briefly, immediately after donation, stools are placed in normal saline, blended, and sieved to remove particulate matter. Samples are then differentially centrifuged to isolate a bacterial pellet. The bacterial pellet is suspended in normal saline (containing 15% glycerol – a cryoprotectant) at 0.5 g wet weight/ml before being dispensed into size 0 DRcaps™ capsules (Capsugel Inc, Sydney, Australia). The size 0 capsules are closed and secondarily sealed in size 00 DRcaps™ capsules. These capsules mask taste, odour, and visual appearance, and are designed to remain intact during passage through the stomach, delivering their contents to the intestine[38-40]. Capsules are stored frozen at -80°C.

The use of low-speed centrifugation to pellet the bacterial cells is a feature of this methodology that reduces the risk of having free viruses[41] included into the treatment capsules. Storage (-80°C, <175 days[18 19]) of microbiome capsules provides time to complete rigorous safety testing using microbiological and microscopic analyses.

Randomisation, allocation, and blinding

Eligible participants will be randomised in a 1:1 ratio to either treatment or placebo group, stratified by sex, using block randomisation with variable block sizes of 2 and 4[42]. Randomisation sequences will be computer generated, and overseen by the biostatistician. Researchers and participants will be blinded to capsule contents, both of which (placebo and gut microbiome) look identical (white).

There are three steps in the blinding and allocation process. First, the independent research nurse allocates the recipient to group A or B using the randomisation sequence. Second, the placebo and treatment capsule packs each have a unique code (assigned by the technician who encapsulated them). Lastly, the independent research nurse allocates the pack according to the unique code associated with the randomisation sequence.

To maintain the integrity of the trial evaluation, statistical analyses will be performed at the completion of the study on encoded data (*i.e.* Group A *vs* Group B), so that the biostatistician will be blinded to treatment allocation. Recipients will be asked if they are able to identify the contents of capsules taken (*i.e.* placebo or gut microbiome) at 6 weeks and 26 weeks. The effectiveness of treatment blinding will be assessed using the Bang's blinding index[43]. Blinding success will be determined by the thresholds of Moroz et al.[44]: unblinded (BBI ≥ 0.2); random guesses ($-0.2 < \text{BBI} < 0.2$); or opposite guesses (BBI ≤ -0.2).

Recipients will be unblinded in the case of any serious adverse events. These include on-going gastrointestinal bleeding, severe vomiting and/or diarrhoea, treatment related systemic infection, treatment related severe allergic reaction, coma, collapse and death. Unblinding will be done by an independent researcher who did not have any prior contact with the recipient, who will be able to determine the individual's treatment allocation.

Study intervention

All recipients will undergo bowel cleansing prior to treatment using an oral solution containing 70 g of Glycoprep-C® (active ingredient macrogol 3350) (Fresenius Kabi Australia Pty Ltd., Mount Kuring-gai, Australia). Bowel cleansing reduces gut microbial population by 31-fold and markedly reduces bacterial diversity[45]. This procedure was used in a pilot study of gut microbiome transfer in adults with type 2 diabetes[20]. Diminishing the undesirable microbial community means that the donor bacteria are more likely to become established in the recipient's bowel[20].

Recipients will be advised to take the Glycoprep-C® solution between 4 pm and 6 pm the day before the treatment begins. It is expected that watery stools will follow for several hours to achieve bowel cleansing. Recipients will attend clinic early next morning, when each recipient in the placebo group will ingest saline capsules, while those in the treatment group will receive gut microbiome capsules. Each recipient will receive a total of 28 capsules (approximately 14 ml of frozen microbial suspension or saline) administered over two consecutive mornings under direct supervision from research staff[18], specifically 16 capsules in the first morning and 12 capsules in the second morning. Recipients will be fasting overnight for at least 8 hours prior to taking each set of capsules at clinic in the following morning. Capsules will be stored at -80˚C, and later transferred into a freezer at -30°C in the morning of administration. Immediately before administration, treatment capsules will be placed onto gel packs at 4°C to prevent harm to recipients upon swallowing. After treatment, all recipients will remain fasting for another 2 hours. Recipients will be advised not to change their diet, physical activity, and behaviour during the trial. All recipients will receive the same number of capsules from the four same-sex donors

to standardized treatment and to ensure that overall donor microbiome diversity is increased and delivered in a reproducible fashion.

Data collection and follow-up

Timing of assessments

Recipients will be assessed four times over the course of the study: at baseline, 6 weeks, 12 weeks, and 26 weeks. Longitudinal follow-up over 26 weeks will establish the duration of the effect. The specific assessments that will be carried out at each time point are outlined in Table 3. Treatment (*i.e.* intake of capsules) will be administered within a week of the baseline assessment.

Clinical assessments will start between 07:00 am and 09:00 am at the Maurice & Agnes Paykel Clinical Research Unit (Liggins Institute, University of Auckland), after an overnight fast and no strenuous activity over the previous 24 hours.

All the recipients will be contacted and reminded of their follow-up visits via emails and text messages. Any recipient having difficulties to attend their assessment visit will be given the option to re-schedule it to a suitable time.

Insulin sensitivity and other blood tests

Insulin sensitivity will be assessed in all recipients using the Matsuda index from a 75-g oral glucose tolerance test (OGTT)[46]. Blood samples will be collected at -10, 0, 30, 60, 90, and 120 minutes for glucose and insulin measurements. The Matsuda index is highly correlated with the hyperinsulinaemic euglycaemic clamp (the gold-standard assessment of insulin sensitivity[47]) and has excellent reproducibility during multiple measures[48]. Other markers of glycaemic control will also be measured, namely homeostasis model assessment of insulin resistance (HOMA-IR)[49] and glycated haemoglobin (HbA1c).

Other blood tests

Fasting blood samples will be taken during the insulin sensitivity assessment to measure a number of other parameters. These will include markers of metabolic syndrome, such as uric acid, high-sensitivity C-reactive protein (hsCRP), and fasting lipids (*i.e.* total cholesterol, high-density lipoprotein cholesterol [HDL-C], low-density lipoprotein cholesterol [LDL-C], and triglycerides). Liver function will be

assessed by measurement of gamma-glutamyl transferase (GGT), alkaline phosphatase (ALP), alanine aminotransferase (ALT), and aspartate transaminase (AST).

Anthropometry and body composition

Height will be measured to the nearest mm using a Harpenden stadiometer. Recipients will be asked to undo any hairstyle that might interfere with the measurements; they will stand with their feet together, with their back straight, and their heels in the same upright plane as the back of the head. Gentle upward traction on the mastoid process will be applied to straighten out the spine. Weight will be measured on a weighing scale (WM206, Wedderburn, Auckland, New Zealand) to the nearest 10 g. For weight measurements, the scale will be placed on a solid level floor, with recipients stepping on it with both feet at its centre. Both height and weight will be measured three times and the median value used for analysis; recipients will also be asked to remove shoes and bulky clothing, and to empty their pockets of any objects. Both the scale and stadiometer at our clinical research unit are checked on a weekly basis using the appropriate standards.

BMI will be calculated and transformed into standard deviations scores (SDS) adjusted for age and sex, based on WHO standards [50]. Waist and hip circumferences will be measured as per guidelines from the World Health Organization[51]. Both the waist and hip circumference measurements will be performed three times and the median value used for analysis. Both measurements will be made to the nearest mm with a standard measuring tape parallel to the floor, which is placed snugly around the recipient but without compressing the skin[52]. Body composition will be assessed using whole-body dual-energy X-ray absorptiometry (DXA, Lunar Prodigy™ and Lunar iDXA ™, GE Medical Systems, Chicago, Illinois, USA). Recipients will have all longitudinal body composition data collected on the same device.

Blood pressure

Clinic resting systolic and diastolic blood pressures will be measured at all assessments using the same oscillometric digital blood pressure monitor (ri-champion® N; Riester, Jungingen, Germany) with an appropriately-sized cuff on the extended non-dominant arm. All measurements will be recorded on each recipient while seated and after a 5-minute rest. Blood pressure will be measured three times, and the median value used for analysis.

In addition, 24-hour ambulatory blood pressure monitoring will be performed at baseline and at 6 weeks, using an oscillometric device (Spacelabs 90217; Spacelabs Medical Inc, Redmond, Washington, USA) on the non-dominant arm. Over a 24-hour period, blood pressure will be measured every 20

minutes when the recipients are expected to be awake, and every 30 minutes when they are likely to be asleep (based on self-reported information). Recipients will be asked to record the time they go to bed and the time they wake up over the period of monitoring, so that waking and sleeping times can be more accurately identified.

Dietary intake

A dietary record describing all foods and fluids consumed over three days will be collected at the 6-week assessment. Recipients will be asked to describe all foods and fluids consumed in detail including brand names, types of foods (e.g. low fat), and cooking methods. Quantities will be described using standard household measures, as well as the information from food labels (where appropriate). Recipients will be provided with standardized instructions for completing the dietary record by a trained investigator, who will also review individual records with recipients to clarify errors, omissions, questionable entries, or unclear descriptions. These dietary records will be entered into FoodWorks software (v9.0, Xyris Software, Brisbane, Australia) by a trained investigator.

The New Zealand Adolescent Food Frequency Questionnaire (NZAFFQ)[53] will be administered at baseline and weeks 6, 12, and 26. The NZAFFQ was developed for and validated in New Zealand adolescents aged 14 to 18 years[53].

Physical activity levels

These will be measured using two questionnaires:

• International Physical Activity Questionnaire (IPAQ)[54] – it covers four domains of physical activity, namely work-related, transportation, housework/gardening, and leisure time.

• Adolescent Sedentary Activity Questionnaire (ASAQ) [55] – it covers a number of sedentary activities across five categories (small screen recreation, education, travel, cultural activities, and social activities).

Health-related quality of life

This will be assessed using:

• EPOCH Measure of Adolescent Well-Being[56] – it provides an assessment of five positive psychological characteristics (engagement, perseverance, optimism, connectedness, and happiness).

• Pediatric Quality of Life Inventory (PedsQL)[57] – we will adopt only the teen and young adult self-reports (*i.e.* not the parent-proxy), which assess problems over the preceding month relating to physical, emotional, social, and school functioning.

In addition, we will assess symptoms of irritable bowel syndrome[58] and bowel movements using the Birmingham IBS symptom questionnaire and bowel movements questionnaire respectively. The Birmingham IBS symptom questionnaire is a self-administered 11-item symptom questionnaire that is scored using the Rome II criteria[58]. The bowel movement questionnaire was designed for this trial to assess and monitor changes pre and post treatment.

Gut microbial composition

Sample collection will be performed at baseline prior to treatment and at 6 weeks, 12 weeks and 26 weeks post-treatment. Briefly, the participant will be given the bedpan liner (Onelink). They will be asked to: i) pass urine into the toilet prior to placing the tray on the toilet seat; ii) pass the stools; iii) cover the tray and leave it in the bathroom for immediate collection by a research team member. Using a small spatula, samples will be collected from three different areas of the stool (proximal, middle, and distal) and inserted into specimen containers (Onelink). The specimen containers will be immediately placed on ice and taken to the laboratory where they will be frozen and stored at -80°C. DNA and RNA extraction will be completed within 5 days of donation. Time to processing will be recorded.

Note that we will advise participants to try not to have a bowel movement in the morning prior to their visit, having it in the clinic instead. For those participants who are unable to produce a stool sample during their visit, they will be provided with a stool collection kit to take home and detailed instructions on how to collect the stool sample. This kit is made up of: i) instructions on how to use the stool collection kit; ii) specimen container; and iii) bedpan liner. Once the stool has been collected in the home environment, the specimen container it should be immediately placed into their home freezer, and kept there until it is delivered to the research team.

All extractions will be performed using Qiagen-AllPrep DNA/RNA mini kit®, due to variation in extraction efficiencies with the different kits [59]. However, once the DNA or RNA is extracted and archived, we will have a relatively stable record of the composition and activity of the flora.

Frozen faeces (~200 mg; weights will be recorded) will be subsampled from original faecal samples. All DNA and RNA isolations will be performed in a disinfected class II hood at room temperature. Briefly, stool samples will be incubated (10 min, room temperature) with vortexing (30 sec every 2

minutes) and treated with RLT Plus buffer (1.2mL; Qiagen) and 12µL beta-mercaptoethanol (Sigma-Aldrich). Acid-washed glass beads [1 ml; ≤106 µm (-140 U.S. sieve) (Sigma-Aldrich)] will be added to each sample and vortexed (10 min) on a TissueLyzer II (Qiagen). The supernatant will be removed and added to a QIAshredder spin column (Qiagen) and centrifuged (9000 rpm, 2 min, room temperature). The eluent will be added to an AllPrep DNA (Qiagen) spin column and centrifuged (30 sec, 14000 rpm, room temperature). The eluent and AllPrep DNA spin columns will be used for RNA and DNA extraction, respectively, according to the manufacturer's instructions. Finally, DNA and RNA will be eluted with EB buffer and RNase-free water, respectively, and aliquots stored at -80°C for downstream mixed omics analysis.

A series of blank samples (sterile saline) will be extracted in parallel to sample extractions to enable contamination testing. We will also extract ZymoBIOMICS™ Microbial Community Standard I (Even, Cellular Mix; Catalog #D6300) to determine potential bias in the extraction process.

For 16S amplicon sequencing, library preparation will be performed using an Illumina platform by a commercial provider (to be determined) using standard protocols for the SV3-4 region. Shotgun metagenomics sequencing will be performed by a commercial provider (to be determined).

All raw sequencing files will be cleaned to remove adaptors and primer sequences, and trimmed for sequence quality (Phred score<30).

Longitudinal analysis of gut microbiome data (i.e. change in alpha and beta diversity from baseline to 26 weeks in treatment and placebo group) will be performed on Qiime2 (version 2018.4 or later) using default parameters [60]. PERMANOVA and Multivariate Association with Linear Models using MaAsLin (version 0.0.4; or later) [61] will be used to identify any significant differences in gut microbial communities and structure between treatment groups.

Metagenomic sequencing data will be analysed using default parameters of the HMP Unified Metabolic Analysis NEtwrok (HuMAnN2) (version 2; or later) [62] after removal of short reads (minimum length 50 bases, trimmomatic version 0.33 or later [63]) and human sequences using BMTagger [64]. MaAsLin (version 0.0.4; or later) [61] will be used to identify significant associations between microbial compositions, metabolomics data, and microbial functions.

Safety monitoring

An independent safety monitoring committee has been established. All recipients are advised to remain under supervision in the clinical research unit for one hour after initial treatment and we will adopt

robust exclusion and screening criteria for donors (as previously described). In addition, recipients' data will be monitored by the research team and the safety committee throughout the study for any adverse events, in particular gastrointestinal symptoms and possible allergies. All potential adverse events will be recorded. If any recipient suffers harm as a result of trial participation, they will be eligible to apply for compensation from the Accident Compensation Cooperation (ACC), which is a compulsory insurance cover for personal injury for everyone in New Zealand.

If any concerns are identified during screening or clinical assessment of donors or recipients, further clinical evaluation and/or investigation will be immediately undertaken. If concerns are identified during the study, the recipient will be withdrawn if this is thought to be in their best interest.

Outcome measures

Primary outcome

- BMI SDS at 6 weeks.

Secondary outcomes
- BMI SDS at 12, and 26 weeks
- total body fat percentage (from DXA) at 6, 12, and 26 weeks
- insulin sensitivity at 6, 12, and 26 weeks
- gut microbial composition at 6, 12, and 26 weeks
- liver function at 6, 12, and 26 weeks
- lipid profile at 6, 12, and 26 weeks
- inflammatory markers [uric acid, high-sensitivity C-reactive protein (hsCRP)] at 6, 12, and 26 weeks
- blood pressure at 6, 12, and 26 weeks
- health-related quality of life at 6, 12, and 26 weeks
- IBS symptoms at 6, 12, and 26 weeks
- bowel movements at 6, 12, and 26 weeks

Sample size and power calculation

Power calculation was based on data from a cohort of 50 obese adolescents in Australia aged 14–18 years, with a pooled mean BMI SDS of 2.5 and standard deviation of 0.27 at baseline[65]. A study with 32 recipients per group will have 80% power at 5% significance level (two-sided) to detect a group difference of 0.19 in BMI SDS at 6 weeks after gut microbiome transfer, which is equivalent to a

difference in weight of approximately 2 kg. To account for an approximate 20% loss to follow-up, we aim to recruit 40 treatment and 40 control recipients.

Data management

All data collected will be entered and stored in password-protected web-based platforms. Rules for data validation will be in place to minimize human error, and all data entered by members of the research team will be double-checked by the database administrator to ensure accuracy of stored records. Only the researchers involved in the trial will have access to the final trial dataset.

Statistical analyses

Treatment evaluation will be performed on the principle of intention to treat, using data collected from all randomised recipients. Baseline demographics and clinical characteristics of recipients will be summarised by randomised group. The distribution of outcome measures will be first evaluated at scheduled visits using descriptive statistics. Generalised linear regression models will be used to assess the main treatment effects between groups, adjusting for the baseline outcome value [66] and sex (stratification factor). Model-adjusted estimates and the differences between the two groups will be calculated with 95% confidence intervals. Random effects mixed models will be used to evaluate the outcomes measured repeatedly over time, controlling for correlated data collected from the same recipient. Planned subgroup analysis by sex will be conducted on primary and secondary outcomes to evaluate the consistency of main treatment effects in males and females, by including an interaction term between sex and treatment group in the main model. If a significant interaction effect is found, separately subgroup analyses will be conducted to estimate the treatment effects in specific subgroups.

Missing data on the primary outcome will be imputed using multiple imputations, which create multiple imputed datasets for the incomplete outcome variable that are analyzed using same regression models and combined for one inference. The Markov chain Monte Carlo (MCMC) method will be used to produce the parameter estimates, assuming the data are from a multivariate normal distribution and are missing at random. The SAS procedure, PROC MI, will be used which runs 200 iterations of the algorithm before selecting the first completed data set, and then allows 100 iterations between each successive data set. The default minimum number of imputations is 5, and we plan to run 30 to allow for both within and between imputation variances.

Per-protocol analyses will be carried out on those recipients without major protocol violations. A protocol deviation form will be used to record all major protocol deviations, and reviewed in a blinded fashion by the trial steering group prior to final data lock. The per-protocol population will be analysed

using same regression models as the primary intention-to-treat (ITT) population to test the robustness of main trial findings.

Our secondary analyses will include the examination of potential effects of diet (e.g. fibre intake) and physical activity levels on study outcomes. Data analyses will be performed in SAS v.9.4 (SAS Institute, Cary, NC, USA), SPSS v25 (IBM Corp, Armonk, NY, USA), and/or Minitab v.16 (Pennsylvania State University, State College, PA, USA). All statistical tests will be two-sided at $p<0.05$, with no adjustments for multiple comparisons. The CONSORT 2010 guidelines will be followed in reporting the main trial results.

Study status

The recruitment of recipients for the trial began in Oct 2017. It is expected that the study will be completed in mid-2019.

Patient and public involvement

Public input into the study design was provided in open meetings by the Northern A Health and Disability Ethics Committee, whose membership includes both clinical and lay persons, as well as Māori representatives (New Zealand indigenous people). Information on the trial was subsequently made available on social media platforms (e.g. Facebook), which allowed participants to read and contact the researchers if they wanted to participate. Participants were not involved in the development, recruitment of other participants, or conduct of the trial. All recipients will be asked about any possible adverse effects of treatment at specific time points throughout the trial; if any serious adverse effects are reported, a thorough follow-up will be conducted to investigate the incident. After completion of data analyses, all recipients will receive information about their individual results.

ETHICS AND DISSEMINATION

Ethics approval for this study was granted in November 2016 by the Northern A Health and Disability Ethics Committee (Ministry of Health, New Zealand; 16/NTA/172). Involvement in this trial will be entirely voluntary. If a recipient agrees to take part, they will be free to withdraw from the study at any time. In addition, the participant will be withdrawn if the research team believes their ongoing involvement in the study is not in their best interest. Donors and recipients will be required to provide written informed consent prior to participation in the study. The Ethics Committee requires that a yearly progress report is submitted, which must disclose any protocol violations.

Clinical and biochemical data will be entered into secure databases protected by passwords, with access restricted to investigators. Recipients and caregivers will be informed of incidental findings on unrecognized conditions (*e.g.* diabetes), with further medical follow-up arranged. Importantly, if at the end of the trial we find that gut microbiome transfer leads to a statistically significant improvement in key health outcomes, the treatment will be offered to all recipients who received placebo. Recipients and caregivers will also be provided with information on individual results.

Communication to the scientific community will be through high-profile international research meetings, as well as relevant national and regional meetings. We aim to publish findings in high-impact peer-reviewed international journals. Further, the research team will communicate the findings to the general public in New Zealand and overseas through our institution's Communications Manager. Relevant findings will be shared with the community in a culturally appropriate manner.

REGISTRATION DETAILS

This study is registered with the Australian New Zealand Clinical Trials Registry (ACTRN: ACTRN12615001351505). In addition, the Universal Trial Number (UTN), World Health Organization, has been obtained (U1111-1176-6753).

Author contributions: WSC, JMO, JGBD, KSWL, BBA, VC, DJH, DMS, TNJ, YJ, KLB, CAC, WS, and TV contributed to the conception and design of the study. KSWL, JGBD, WSC, JMO, TNJ, BBA, and VC drafted the protocol with input from all other authors.

Acknowledgements: We thank Wendy Ranson, Yara Gerber, and Janene Biggs for their valuable assistance during the development of this trial, and Khan Safayet Hossin for help with the database.

Funding: This research received funding from the Rockfield Trust and A Better Start – National Science Challenge. VC was supported by a Long-Term Research Fellowship from the European Society for Paediatric Endocrinology (ESPE), TNJ received a University of Auckland Scholarship, and BBA is supported by a Maurice Paykel Research Fellowship.

Competing interests: None declared.

REFERENCES

1. World Health Organization. Ending Childhood Obesity Report of The Commission on Ending Childhood Obesity. Geneva, 2016.

2. The NS, Suchindran C, North KE, *et al.* Association of adolescent obesity with risk of severe obesity in adulthood. *JAMA* 2010;304:2042-7.

3. Guo SS, Roche AF, Chumlea WC, *et al.* The predictive value of childhood body mass index values for overweight at age 35 y. *Am J Clin Nutr* 1994;59:810-9.

4. Halfon N, Larson K, Slusser W. Associations between obesity and comorbid mental health, developmental, and physical health conditions in a nationally representative sample of US children aged 10 to 17. *Academic Pediatr* 2013;13:6-13.

5. Engeland A, Bjorge T, Sogaard AJ, *et al.* Body mass index in adolescence in relation to total mortality: 32-year follow-up of 227,000 Norwegian boys and girls. *Am J Epidemiol* 2003;157:517-23.

6. Twig G, Yaniv G, Levine H, *et al.* Body-mass index in 2.3 million adolescents and cardiovascular death in adulthood. *N Engl J Med* 2016;374:2430-40.

7. Burcelin R, Serino M, Chabo C, *et al.* Metagenome and metabolism: the tissue microbiota hypothesis. *Diabetes Obes Metab* 2013;15:61-70.

8. Musso G, Gambino R, Cassader M. Obesity, diabetes, and gut microbiota: the hygiene hypothesis expanded? *Diabetes Care* 2010;33:2277-84.

9. Backhed F, Ding H, Wang T, *et al.* The gut microbiota as an environmental factor that regulates fat storage. *Proc Natl Acad Sci U S A* 2004;101:15718-23.

10. Turnbaugh PJ, Backhed F, Fulton L, *et al.* Diet-induced obesity is linked to marked but reversible alterations in the mouse distal gut microbiome. *Cell Host Microbe* 2008;3:213-23.

11. Backhed F, Manchester JK, Semenkovich CF, *et al.* Mechanisms underlying the resistance to diet-induced obesity in germ-free mice. *Proc Natl Acad Sci U S A* 2007;104:979-84.

12. Turnbaugh PJ, Ridaura VK, Faith JJ, *et al.* The effect of diet on the human gut microbiome: a metagenomic analysis in humanized gnotobiotic mice. *Sci Transl Med* 2009;1:6ra14.

13. Dumas ME, Barton RH, Toye A, *et al.* Metabolic profiling reveals a contribution of gut microbiota to fatty liver phenotype in insulin-resistant mice. *Proc Natl Acad Sci U S A* 2006;103:12511-6.

14. Ma J, Zhou Q, Li H. Gut microbiota and nonalcoholic fatty liver disease: Insights on mechanisms and therapy. *Nutrients* 2017;9

15. Evans JM, Morris LS, Marchesi JR. The gut microbiome: the role of a virtual organ in the endocrinology of the host. *J Endocrinol* 2013;218:R37-47.

16. Hota SS, Achonu C, Crowcroft NS, *et al.* Determining mortality rates attributable to *Clostridium difficile* infection. *Emerg Infect Dis* 2012;18:305-7.

17. van Nood E, Vrieze A, Nieuwdorp M, *et al.* Duodenal infusion of donor feces for recurrent Clostridium difficile. *N Engl J Med* 2013;368:407-15.

18. Youngster I, Russell GH, Pindar C, *et al.* Oral, capsulized, frozen fecal microbiota transplantation for relapsing *Clostridium difficile* infection. *JAMA* 2014;312:1772-8.

19. Hirsch BE, Saraiya N, Poeth K, *et al.* Effectiveness of fecal-derived microbiota transfer using orally administered capsules for recurrent Clostridium difficile infection. *BMC Infect Dis* 2015;15:191.

20. Vrieze A, Van Nood E, Holleman F, *et al.* Transfer of intestinal microbiota from lean donors increases insulin sensitivity in individuals with metabolic syndrome. *Gastroenterology* 2012;143:913-6 e7.

21. Weingarden A, Gonzalez A, Vazquez-Baeza Y, *et al.* Dynamic changes in short- and long-term bacterial composition following fecal microbiota transplantation for recurrent Clostridium difficile infection. *Microbiome* 2015;3:10.

22. Walker AW, Parkhill J. Microbiology. Fighting obesity with bacteria. *Science* 2013;341:1069-70.

23. Kootte RS, Levin E, Salojarvi J, *et al.* Improvement of insulin sensitivity after lean donor feces in metabolic syndrome is driven by baseline intestinal microbiota composition. *Cell Metab* 2017;26:611-19.e6.

24. Alang N, Kelly CR. Weight gain after fecal microbiota transplantation. *Open Forum Infect Dis* 2015;2:ofv004.

25. Ridaura VK, Faith JJ, Rey FE, *et al.* Gut microbiota from twins discordant for obesity modulate metabolism in mice. *Science* 2013;341:1241214.

26. Hill C, Guarner F, Reid G, *et al.* Expert consensus document. The International Scientific Association for Probiotics and Prebiotics consensus statement on the scope and appropriate use of the term probiotic. *Nat Rev Gastroenterol Hepatol* 2014;11:506-14.

27. Park S, Bae JH. Probiotics for weight loss: a systematic review and meta-analysis. *Nutr Res* 2015;35:566-75.

28. Sun J, Buys N. Effects of probiotics consumption on lowering lipids and CVD risk factors: A systematic review and meta-analysis of randomized controlled trials. *Ann Med* 2015;47:430-40.

29. Liou AP, Paziuk M, Luevano JM, Jr., *et al.* Conserved shifts in the gut microbiota due to gastric bypass reduce host weight and adiposity. *Sci Transl Med* 2013;5:178ra41.

30. Kassam Z, Lee CH, Yuan Y, *et al.* Fecal microbiota transplantation for *Clostridium difficile* infection: systematic review and meta-analysis. *Am J Gastroenterol* 2013;108:500-8.

31. Colman RJ, Rubin DT. Fecal microbiota transplantation as therapy for inflammatory bowel disease: a systematic review and meta-analysis. *J Crohns Colitis* 2014;8:1569-81.

32. Chan AW, Tetzlaff JM, Altman DG, *et al.* SPIRIT 2013 statement: defining standard protocol items for clinical trials. *Ann Intern Med* 2013;158:200-7.

33. Markle JG, Frank DN, Mortin-Toth S, *et al.* Sex differences in the gut microbiome drive hormone-dependent regulation of autoimmunity. *Science* 2013;339:1084-88.

34. New Zealand Blood Service. Detailed eligibility criteria. 2015.

35. Bibby K, Peccia J. Identification of viral pathogen diversity in sewage sludge by metagenome analysis. *Environ Sci Technol* 2013;47:1945-51.

36. Lacy BE, Patel NK. Rome criteria and a diagnostic approach to irritable bowel syndrome. *J Clin Med* 2017;6:E99.

37. Vanner SJ, Depew WT, Paterson WG, *et al.* Predictive value of the Rome criteria for diagnosing the irritable bowel syndrome. *Am J Gastroenterol* 1999;94:2912-7.

38. Marzorati M, Possemiers S, Verhelst A, *et al.* A novel hypromellose capsule, with acid resistance properties, permits the targeted delivery of acid-sensitive products to the intestine. *LWT Food Sci Technol* 2015;60:544-51.

39. Miller DS, Parsons AM, Bresland J, *et al.* A simple and inexpensive enteric-coated capsule for delivery of acid-labile macromolecules to the small intestine. *J Zhejiang Univ-Sci B* 2015;16:586-92.

40. Amo R. DRcaps® capsules achieve delayed release properties for nutritional ingredients in human clinical study: Capsugel Inc, 2014.

41. Bourdin G, Schmitt B, Marvin Guy L, *et al.* Amplification and purification of T4-like *Escherichia coli* phages for phage therapy: from laboratory to pilot scale. *Appl Environ Microbiol* 2014;80:1469-76.

42. Sealed Envelope Ltd. Create a blocked randomisation list 2016 [Available from: https://www.sealedenvelope.com/simple-randomiser/v1/lists accessed 3 Mar 2017.

43. Bang H, Ni L, Davis CE. Assessment of blinding in clinical trials. *Control Clin Trials* 2004;25:143-56.

44. Moroz A, Freed B, Tiedemann L, *et al.* Blinding measured: a systematic review of randomized controlled trials of acupuncture. *Evid Based Complement Alternat Med* 2013;2013:708251.

45. Jalanka J, Salonen A, Salojarvi J, *et al.* Effects of bowel cleansing on the intestinal microbiota. *Gut* 2015;64:1562-8.

46. Matsuda M, DeFronzo RA. Insulin sensitivity indices obtained from oral glucose tolerance testing: comparison with the euglycemic insulin clamp. *Diabetes Care* 1999;22:1462-70.

47. Lorenzo C, Haffner SM, Stančáková A, *et al.* Relation of direct and surrogate measures of insulin resistance to cardiovascular risk factors in nondiabetic finnish offspring of type 2 diabetic individuals. *J Clin Endocrinol Metab* 2010;95:5082-90.

48. Maki KC, Rains TM, Dicklin MR, *et al.* Repeatability of indices of insulin sensitivity and secretion from standard liquid meal tests in subjects with type 2 diabetes mellitus or normal or impaired fasting glucose. *Diabetes Technol Ther* 2010;12:895-900.

49. Wallace TM, Levy JC, Matthews DR. Use and abuse of HOMA modeling. *Diabetes Care* 2004;27:1487-95.

50. de Onis M, Onyango AW, Borghi E, *et al.* Development of a WHO growth reference for school-aged children and adolescents. *Bull World Health Organ* 2007;85:660-7.

51. World Health Organization. Waist circumference and waist-hip ratio: report of a WHO expert consultation, Geneva, 8–11 December 2008. Geneva, 2008.

52. National Center for Health Statistics. National Health and Nutrition Examination Survey III - Body Measurements (Anthropometry). Rockville, MD, USA: Centers for Disease Control and Prevention, 1988.
53. Wong JE, Parnell WR, Black KE, *et al.* Reliability and relative validity of a food frequency questionnaire to assess food group intakes in New Zealand adolescents. *Nutr J* 2012;11:65.
54. Hagstromer M, Oja P, Sjostrom M. The International Physical Activity Questionnaire (IPAQ): a study of concurrent and construct validity. *Public Health Nutr* 2006;9:755-62.
55. Hardy LL, Booth ML, Okely AD. The reliability of the Adolescent Sedentary Activity Questionnaire (ASAQ). *Prev Med* 2007;45:71-4.
56. Kern ML, Benson L, Steinberg EA, *et al.* The EPOCH Measure of Adolescent Well-Being. *Psychol Assess* 2016;28:586-97.
57. Varni JW, Seid M, Knight TS, *et al.* The PedsQL 4.0 Generic Core Scales: sensitivity, responsiveness, and impact on clinical decision-making. *J Behav Med* 2002;25:175-93.
58. Roalfe AK, Roberts LM, Wilson S. Evaluation of the Birmingham IBS symptom questionnaire. *BMC Gastroenterol* 2008;8:30.
59. Claassen S, du Toit E, Kaba M, *et al.* A comparison of the efficiency of five different commercial DNA extraction kits for extraction of DNA from faecal samples. *J Microbiol Methods* 2013;94:103-10.
60. Caporaso JG, Kuczynski J, Stombaugh J, *et al.* QIIME allows analysis of high-throughput community sequencing data. *Nature Methods* 2010;7:335.
61. Morgan XC, Tickle TL, Sokol H, *et al.* Dysfunction of the intestinal microbiome in inflammatory bowel disease and treatment. *Genome Biol* 2012;13:R79.
62. Franzosa EA, McIver LJ, Rahnavard G, *et al.* Species-level functional profiling of metagenomes and metatranscriptomes. *Nature Methods* 2018:1.
63. Bolger AM, Lohse M, Usadel B. Trimmomatic: a flexible trimmer for Illumina sequence data. *Bioinformatics* 2014;30:2114-20.
64. Rotmistrovsky K, Agarwala R. BMTagger: Best Match Tagger for removing human reads from metagenomics datasets. Key: citeulike:9207261; 2011
65. O'Brien PE, Sawyer SM, Laurie C, *et al.* Laparoscopic adjustable gastric banding in severely obese adolescents: a randomized trial. *JAMA* 2010;303:519-26.
66. Committee for Medicinal Products for Human Use. Guideline on adjustment for baseline covariates in clinical trials. Report EMA/CHMP/295050/2013. London: European Medicines Agency, 2015.
67. Bakken JS, Borody T, Brandt LJ, *et al.* Treating *Clostridium difficile* infection with fecal microbiota transplantation. *Clin Gastroenterol Hepatol* 2011;9:1044-9.

Figure 1. Diagram showing flow of participants (recipients) in the Gut Bugs Trial.

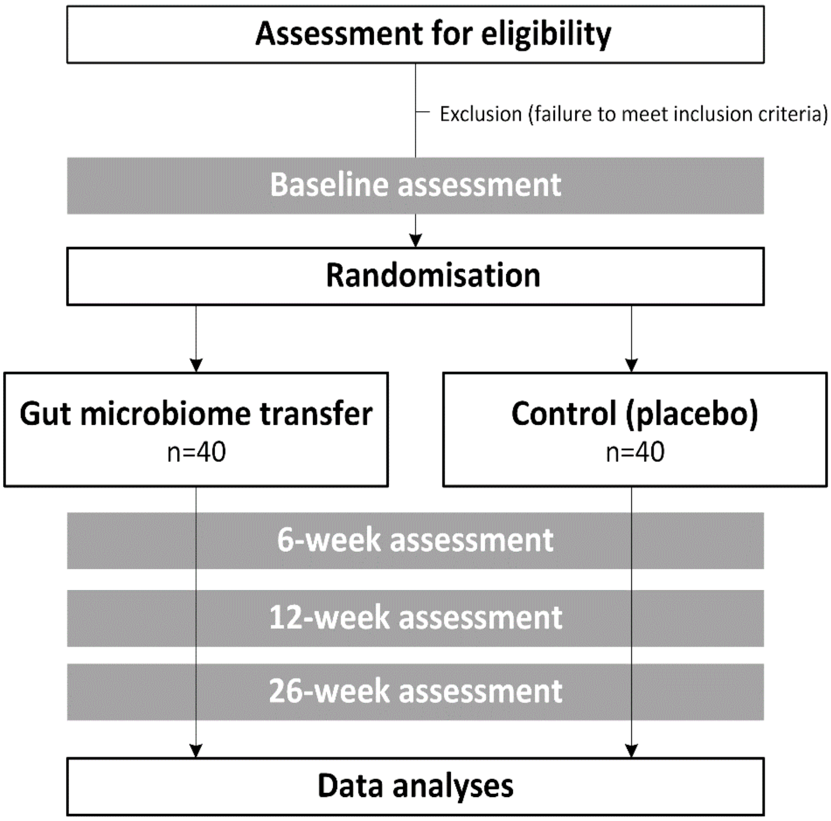

Table 1. Inclusion and exclusion criteria for donors in the Gut Bugs Trial. Exclusion criteria adapted from Youngster et al., Hirsch et al., van Nood et al., and Bakken et al.[17-19,67].

**Inclusion**	• Age 18 to 28 years
	• BMI >18.5 kg/m^2 and <30.0 kg/m^2
	• Total body fat ≤29% for females and ≤19% for males
	• Regular exercise (moderate to vigorous physical activity for at least 3.5 hours per week)
	• Regular bowel habit (at least once every two days)
	• Intake of ≥4 portions of fruit and/or vegetables per day
**Exclusion**	• Any transmissible viral or bacterial pathogens, or intestinal parasites
	• Multidrug-resistant organisms (*e.g.* vancomycin-resistant enterococci, extended-spectrum beta-lactamase-producing Enterobacteriaceae, and carbapenem-resistant Enterobacteriaceae)
	• Gastrointestinal disease (including symptoms of irritable bowel syndrome, inflammatory bowel disease, or coeliac disease)
	• Atopic diseases requiring regular prophylaxis or treatment
	• Current or past history of malignancy
	• Impaired fasting glucose or impaired glucose tolerance
	• Type 1 diabetes, type 2 diabetes, or monogenic diabetes
	• Known dyslipidaemia, hypertension, or metabolic syndrome
	• Regular use of medications known to influence metabolism or the gut microbiome
	• Use of oral antibiotics in the past three months
	• Regular 'binge drinking', *i.e.* consumption of 5 or more standard drinks of alcohol per session, at least once a week
	• Any use of recreational drugs or tobacco
	• Current or past pregnancy
	• Overseas travel in previous 6 months, except for visits to Australia, UK, USA, Canada, Northern Europe, France, and Germany.
	• UK residence in 1980–1996 (due to risk of variant Creutzfeldt-Jakob disease)

Table 2. Inclusion and exclusion criteria for recipients in the Gut Bugs Trial.

**Inclusion**	• Aged 14 to 18 years<br>• BMI ≥30 kg/m^2<br>• Post-pubertal (Tanner stage 5)
**Exclusion**	• Gastrointestinal disease (including inflammatory bowel disease or coeliac disease)<br>• Use of regular medications that may influence weight, metabolism, or the gut microbiome (including oral oestrogen-containing contraceptives, antidepressants, glucose-lowering drugs, diet drugs, as well as inhaled, topical, or oral steroids)<br>• Consumption of probiotics<br>• Type 1 diabetes, type 2 diabetes, or monogenic diabetes<br>• Chronic diseases that could affect the primary outcome (other than obesity-related conditions)<br>• Food allergies<br>• Allergy to macrogol (active ingredient in the bowel preparation product)<br>• Allergy to any over-the-counter medication<br>• No antibiotic usage for three months prior to trial treatment

Table 3. Timing of individual assessments in the Gut Bugs Trial.

		Baseline	6 weeks	12 weeks	26 weeks
**Clinic**	Medical history and exam	✓	✓	✓	✓
	Anthropometry	✓	✓	✓	✓
	DXA	✓	✓	✓	✓
	Clinic blood pressure	✓	✓	✓	✓
	24-h ambulatory blood pressure monitoring	✓	✓	-	-
**Questionnaires**	3-day dietary record	-	✓	-	-
	NZAFFQ	✓	✓	✓	✓
	Birmingham IBS	✓	✓	✓	✓
	Bowel movement questionnaire	✓	✓	✓	✓
	PedsQL	✓	✓	✓	✓
	EPOCH	✓	✓	✓	✓
	IPAQ	✓	✓	✓	✓
	ASAQ	✓	✓	✓	✓
**Laboratory**	Matsuda Index	✓	✓	✓	✓
	HOMA-IR	✓	✓	✓	✓
	HbA1c	✓	✓	✓	✓
	Fasting lipid profile	✓	✓	✓	✓
	Liver function tests	✓	✓	✓	✓
	hsCRP and uric acid	✓	✓	✓	✓
**Stool bacteriology**	Gut microbial composition via 16S rRNA amplicon sequencing	✓	✓	✓	✓
	Metagenome	✓	✓	-	-

ASAQ, Adolescent Sedentary Activity Questionnaire; DXA, Dual-energy x-ray absorptiometry; EPOCH, Engagement Perseverance Optimism Connectedness Happiness; HbA1c, glycated haemoglobin; HOMA-IR, homeostasis model assessment of insulin resistance; hsCRP, high-sensitivity C-Reactive Protein; IBS, irritable bowel syndrome; IPAQ, International Physical Activity Questionnaire; NZAFFQ, New Zealand Adolescent food frequency questionnaire; PedsQL, Pediatric Quality of Life Inventory.
