# Supplementary material for: Effects of Fecal Microbiome Transfer in Adolescents With Obesity: The Gut Bugs Randomized Controlled Trial
Source: JAMA Netw Open. 2020 Dec 21;3(12):e2030415. doi: 10.1001/jamanetworkopen.2020.30415 (PMC7753902; doi:10.1001/jamanetworkopen.2020.30415)
Supplement: Supplement 2. — Statistical Analysis Plan [file jamanetwopen-e2030415-s002.pdf]

Statistical Analysis Plan

Trial Registration number	ACTRN12615001351505p
Universal trial number	U1111-1176-6753

Trial Title : Gut Bugs Trial

By: Dr Karen Leong

Date: 10/04/2019

Version: Version 1.0

Protocol: Version 1.0

Funding: Rockfield Trust

A Better Start – National Science Challenge

SIGNATURE PAGE

CLINICIAN

Dr Karen Leong
Liggins Institute – University of Auckland
Auckland – New Zealand
k.leong@auckland.ac.nz

Signature

Date: 10|4|2019

TRIAL STATISTICIAN

Dr José Derraik
Liggins Institute – University of Auckland
Auckland – New Zealand
j.derraik@auckland.ac.nz

Signature

Date: 10/4/2019

SENIOR BIOSTATISTICIAN

Dr Yannan Jiang
Department of Statistics
Liggins Institute – University of Auckland
Auckland – New Zealand
y.jiang@auckland.ac.nz

Signature

Date: 10/4/2019

PRINCIPAL INVESTIGATOR

Prof Wayne Cutfield
Liggins Institute – University of Auckland
Auckland – New Zealand
w.cutfield@auckland.ac.nz

Signature

Date: 10/4/2019

PRINCIPAL INVESTIGATOR

Assoc Prof Justin O'Sullivan
Liggins Institute – University of Auckland
Auckland – New Zealand
justin.osullivan@auckland.ac.nz

Signature

Date: 10/4/19

CO-INVESTIGATOR - MICROBIOME

Dr Tommi Vatanen
Liggins Institute – University of Auckland
Auckland – New Zealand
t.vatanen@auckland.ac.nz

Signature

Date: 10|4|2019

TABLE OF CONTENTS

1 INTRODUCTION 5
1.1 Background 5
1.2 Objectives 5

2 STUDY METHODS 5
2.1 Trial design 5
2.2 Recruitment and eligibility criteria 5
2.2.1 Donors 5
2.2.2 Participants (recipients) 7
2.3 Randomisation, allocation, and blinding 8
2.4 Study intervention 8
2.5 Outcome measures 9
2.6 Sample size and power calculation 9

3 DATA COLLECTION AND MANAGEMENT 10
3.1 Flow of participants 10
3.2 Scheduled assessments 11
3.3 Withdrawals 11
3.4 Research forms and data collection tools 12
3.5 Safety monitoring and evaluation 14
3.6 Outcome variables and definitions 14
3.6.1 Body mass index standard deviation score (BMI SDS) 14
3.6.2 Total body fat percentage (from DXA) 15
3.6.3 Insulin sensitivity 15
3.6.4 Liver function 15
3.6.5 Lipid profile 15
3.6.6 Inflammatory markers 16
3.6.7 Blood pressure 16
3.6.8 Definitions of abnormal outcomes 17
3.6.9 EPOCH Measure of Adolescent Well-Being 19
3.6.10 Pediatric Quality of Life Inventory (PedsQL) 19
3.6.11 IBS symptoms 19
3.6.12 Bowel movements 19
3.6.13 Gut microbial composition 20
3.7 Diet, physical activity, and socioeconomic status 21
3.7.1 IPAQ 21
3.7.2 ASAQ 23
3.7.3 NZAFFQ 23
3.7.4 3-day food diary 23
3.7.5 Socioeconomic status 23

4 STATISTICAL METHODS 23
4.1 Primary outcome analyses 24

4.2 Secondary outcome analyses 25

5 MICROBIOME DATA ANALYSIS 25

5.1 Initial bioinformatics 25

5.2 Metagenomic assembly 26

5.3 Statistical comparisons 26

5.4 Engraftment analysis 28

5.5 Identification of super-donor behavior 28

6 REPORTING OF TRIAL RESULTS 29

6.1 Baseline characteristics 29

6.2 Study outcomes 29

7 REFERENCES 36

1 INTRODUCTION

1.1 Background

This document contains the statistical analysis plan for the Gut Bugs Trial. In brief, this is a randomised double-blind placebo-controlled trial of gut microbiome transfer for the treatment of obesity in adolescents. This document is prepared following the CONSORT guidelines [1,2]

1.2 Objectives

This trial aims to assess the effectiveness of gut microbiome transfer using encapsulated material for the treatment of obesity in adolescents. We aim to assess the effect of gut microbiome transfer on weight, total body fat, insulin sensitivity, metabolic changes, bowel movements and quality of life in these adolescents.

2 STUDY METHODS

2.1 Trial design

A two-arm, double-blind, placebo-controlled, randomised clinical trial with obese adolescents randomly assigned to either treatment (encapsulated gut microbiome) or placebo (encapsulated saline solution), stratified by sex. Eligible participants will be followed for 26 weeks post randomisation (Figure 1). This trial protocol is reported as per the SPIRIT guidelines [3].

2.2 Recruitment and eligibility criteria

2.2.1 Donors

We will recruit 8 donors (4 males and 4 females), as recipients will only receive gut microbiome from donors of the same sex. This is to enhance microbial variability and standardise the treatment via gut microbiome transfer. Donors will be selected based on strict inclusion criteria (Table 1).

Table 1. Inclusion and exclusion criteria for donors in the Gut Bugs Trial.

**Inclusion**	• Age 18 to 28 years
	• BMI >18.5 kg/m^2 and <30.0 kg/m^2
	• Total body fat ≤29% for females and ≤19% for males
	• Regular exercise (moderate to vigorous physical activity for at least 3.5 hours per week)
	• Regular bowel habit (at least once every two days)
	• Intake of ≥4 portions of fruit and/or vegetables per day
**Exclusion**	• Any transmissible viral or bacterial pathogens, or intestinal parasites
	• Multidrug-resistant organisms (*e.g.* vancomycin-resistant enterococci, extended-spectrum beta-lactamase-producing Enterobacteriaceae, and carbapenem-resistant Enterobacteriaceae)
	• Gastrointestinal disease (including symptoms of irritable bowel syndrome, inflammatory bowel disease, or coeliac disease)
	• Atopic diseases requiring regular prophylaxis or treatment
	• Current or past history of malignancy
	• Impaired fasting glucose or impaired glucose tolerance
	• Type 1 diabetes, type 2 diabetes, or monogenic diabetes
	• Known dyslipidaemia, hypertension, or metabolic syndrome
	• Regular use of medications known to influence metabolism or the gut microbiome
	• Use of oral antibiotics in the past three months
	• Regular 'binge drinking', *i.e.* consumption of 5 or more standard drinks of alcohol per session, at least once a week
	• Any use of recreational drugs or tobacco
	• Current or past pregnancy
	• Overseas travel in previous 6 months, except for visits to Australia, UK, USA, Canada, Northern Europe, France, and Germany.
	• UK residence in 1980–1996 (due to risk of variant Creutzfeldt-Jakob disease)

Eligible donors will be identified by word of mouth, the internal email system at the University of Auckland, and social media networks. Potential donors will be given a detailed information sheet about the study that includes a consent form.

To eliminate the risks of transmission of infectious diseases we will use screening procedures equivalent to those used for blood donation in New Zealand[4], and also screen donors for potential faecal pathogens or multidrug-resistant organisms. As part of this regimen, all potential donors will undergo extensive testing for human pathogens, antigens, and antibodies (that indicate exposure to hepatitis A, B, or C viruses, and human immunodeficiency virus), syphilis, *C. difficile*, *Helicobacter pylori*, other bacterial and viral pathogens, multidrug-resistant organisms, as well as intestinal parasites. We will supplement these microbiological tests with characterisation of the gut microbiome through analysis of the metagenome and metatranscriptome[5]. In addition, we will conduct an interview to gather information about behaviours or activities that may exclude them from the trial.

Given evidence that irritable bowel syndrome (IBS) may be related to the gut microbiome, it is important to exclude potential donors who may have IBS. The Rome criteria are an accepted clinical tool to identify individuals with IBS, but they are relatively insensitive so that strict adherence to those criteria would potentially allow for individuals with mild IBS to donate [6]. Therefore, we will screen for IBS using a conservative modification of the Rome criteria, where we define a positive screen as having 3 or more episodes of abdominal pain per month as described in part I of the criteria, as well as an additional symptom as defined in part II[7].

Each donor is expected to produce a wet stool sample weighing 100-150 g. Our preliminary laboratory data indicate that an average stool sample from a donor will generate sufficient gut microbiome material for two same-sex recipients. Stool samples will be collected and immediately processed for encapsulation. Capsules from each sample will be individually coded, so that each recipient will receive an equal number of capsules (n=7) from each of the four same sex donors.

2.2.2 Participants (recipients)

We aim to recruit 80 obese adolescents according to the inclusion and exclusion criteria described in Table 2.

Table 2. Inclusion and exclusion criteria for recipients in the Gut Bugs Trial.

**Inclusion**	• Aged 14 to 18 years<br>• BMI ≥30 kg/m^2<br>• Post-pubertal (Tanner stage 5)
**Exclusion**	• Gastrointestinal disease (including inflammatory bowel disease or coeliac disease)<br>• Use of regular medications that may influence weight, metabolism, or the gut microbiome (including oral oestrogen-containing contraceptives, antidepressants, glucose-lowering drugs, diet drugs, as well as inhaled, topical, or oral steroids)<br>• Consumption of probiotics<br>• Type 1 diabetes, type 2 diabetes, or monogenic diabetes<br>• Chronic diseases that could affect the primary outcome (other than obesity-related conditions)<br>• Food allergies<br>• Allergy to macrogol (active ingredient in the bowel preparation product)<br>• Allergy to any over-the-counter medication<br>• No antibiotic usage for three months prior to trial treatment

Eligible recipients will be recruited via social media, word of mouth, and paediatric endocrinology clinics in Auckland. Potential recipients and caregivers will be given a detailed information sheet about the study that includes a consent form. Consent will be obtained from recipients if they are aged ≥16 years and from their parents if aged <16 years. Younger recipients will also be asked to sign an assent form. All consent and/or assent will be obtained by the researchers prior to the recipient's participation in the trial. All potential and enrolled recipients’ personal information are

recorded and kept in a secure folder and only accessible to the researchers, in order to protect their confidentiality.

2.3 Randomisation, allocation, and blinding

Eligible participants will be randomised in a 1:1 ratio to either treatment or placebo group, stratified by sex, using block randomisation with variable block sizes of 2 and 4[8]. Randomisation sequences will be computer generated, and overseen by the biostatistician. Researchers and participants will be blinded to capsule contents, both of which (placebo and gut microbiome) look identical (white).

There are three steps in the blinding and allocation process. First, the independent research nurse allocates the recipient to group A or B using the randomisation sequence. Second, the placebo and treatment capsule packs each have a unique code (assigned by the technician who encapsulated them). Lastly, the independent research nurse allocates the pack according to the unique code associated with the randomisation sequence.

To maintain the integrity of the trial evaluation, statistical analyses will be performed at the completion of the study after all trial data have been collected. The biostatistician will be blinded to treatment allocation throughout the trial, as well as study investigators. Recipients will be asked if they are able to identify the contents of capsules taken (*i.e.* placebo or gut microbiome) at 6 weeks and 26 weeks. The effectiveness of treatment blinding will be assessed using the Bang's blinding index[9]. Blinding success will be determined by the thresholds of Moroz et al.[10]: unblinded (BBI ≥ 0.2); random guesses (−0.2 < BBI < 0.2); or opposite guesses (BBI ≤ −0.2).

Recipients will be unblinded in the case of any serious adverse events. These include on-going gastrointestinal bleeding, severe vomiting and/or diarrhoea, treatment related systemic infection, and treatment related severe allergic reaction, coma, collapse and death. Unblinding will be done by an independent researcher who did not have any prior contact with the recipient, who will be able to determine the individual's treatment allocation.

2.4 Study intervention

All recipients will undergo bowel cleansing prior to treatment using an oral solution containing 70 g of Glycoprep-C® (active ingredient macrogol 3350) (Fresenius Kabi Australia Pty Ltd., Mount Kuring-gai, Australia). Recipients will be advised to take the Glycoprep-C® solution between 4 pm and 6 pm the day before the treatment begins. It is expected that watery stools will follow for several hours to achieve bowel cleansing. Recipients will attend clinic early next morning, when

each recipient in the placebo group will ingest saline capsules, while those in the treatment group will receive gut microbiome capsules. Each recipient will receive a total of 28 capsules (approximately 14 ml of frozen microbial suspension or saline) administered over two consecutive mornings under direct supervision from research staff, specifically 16 capsules in the first morning and 12 capsules in the second morning. Recipients will be advised not to change their diet, physical activity, and behaviour during the trial.

2.5 Outcome measures

***Primary outcome**:* BMI SDS at 6 weeks

Secondary outcomes

- BMI SDS at 12, and 26 weeks
- total body fat percentage (from DXA) at 6, 12, and 26 weeks
- insulin sensitivity at 6, 12, and 26 weeks
- gut microbial composition at 6, 12, and 26 weeks
- liver function at 6, 12, and 26 weeks
- lipid profile at 6, 12, and 26 weeks
- inflammatory markers [uric acid, high-sensitivity C-reactive protein (hsCRP)] at 6, 12, and 26 weeks
- blood pressure at 6, 12, and 26 weeks
- health-related quality of life at 6, 12, and 26 weeks
- IBS symptoms at 6, 12, and 26 weeks
- bowel movements at 6, 12, and 26 weeks

All outcomes will be analysed at the end of trial, after all the participants completed their 26 weeks follow up visits.

2.6 Sample size and power calculation

Power calculation was based on data from a cohort of 50 obese adolescents in Australia aged 14–18 years, with a pooled mean BMI SDS of 2.5 and standard deviation of 0.27 at baseline [11]. A study with 32 recipients per group will have 80% power at 5% significance level (two-sided) to detect a group difference of 0.19 in BMI SDS at 6 weeks after gut microbiome transfer. To account for an approximate 20% loss to follow-up, we aim to recruit 40 treatment and 40 control recipients.

3 DATA COLLECTION AND MANAGEMENT

3.1 Flow of participants

Figure 1. Diagram showing flow of participants (recipients) in the Gut Bugs Trial [2].

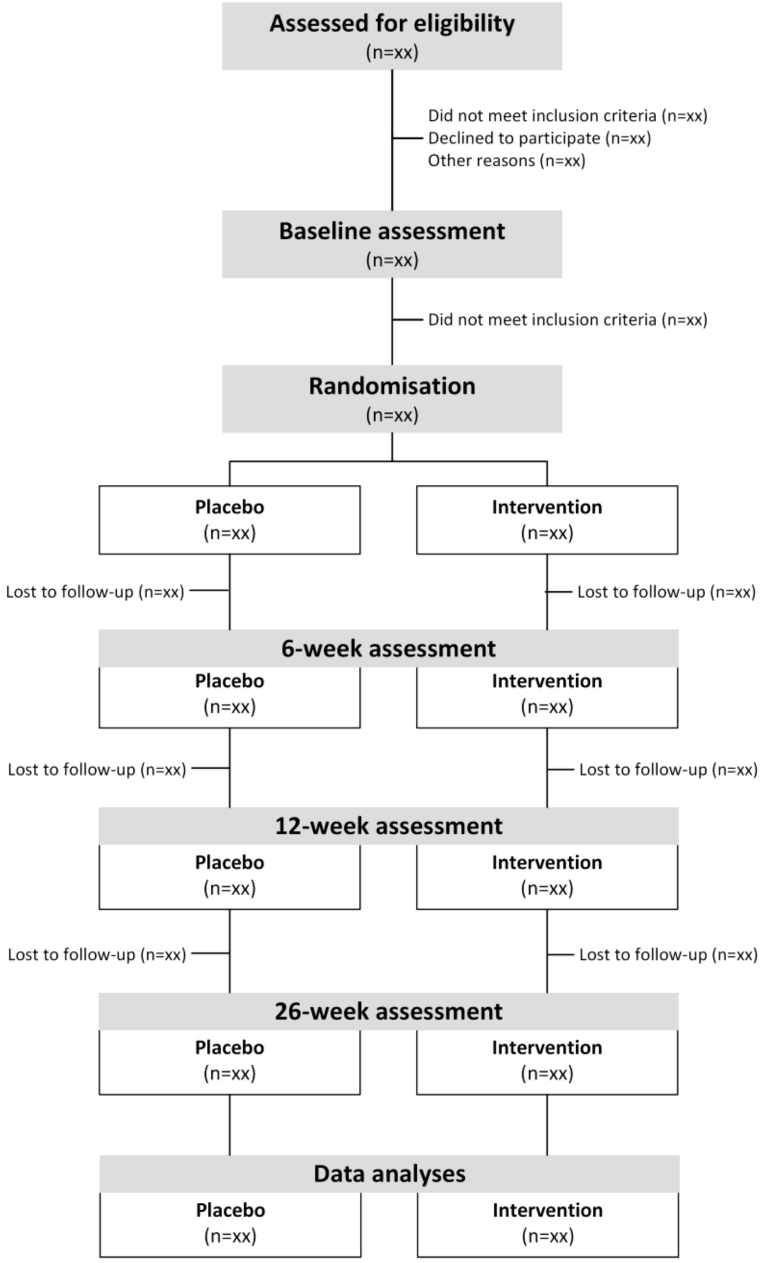

3.2 Scheduled assessments

The timing of scheduled assessments is shown in Table 3.

Table 3. Timing of individual assessments in the Gut Bugs Trial.

		Baseline	6 weeks	12 weeks	26 weeks
**Clinic**	Medical history and exam	✓	✓	✓	✓
	Anthropometry	✓	✓	✓	✓
	DXA	✓	✓	✓	✓
	Clinic blood pressure	✓	✓	✓	✓
	24-h ambulatory blood pressure monitoring	✓	✓	-	-
**Questionnaires**	3-day dietary record	-	✓	-	-
	NZAFFQ	✓	✓	✓	✓
	Birmingham IBS	✓	✓	✓	✓
	Bowel movement questionnaire	✓	✓	✓	✓
	PedsQL	✓	✓	✓	✓
	EPOCH	✓	✓	✓	✓
	IPAQ	✓	✓	✓	✓
	ASAQ	✓	✓	✓	✓
**Laboratory**	Matsuda Index	✓	✓	✓	✓
	HOMA-IR	✓	✓	✓	✓
	HbA1c	✓	✓	✓	✓
	Fasting lipid profile	✓	✓	✓	✓
	Liver function tests	✓	✓	✓	✓
	hsCRP and uric acid	✓	✓	✓	✓
**Stool bacteriology**	Gut microbial composition via 16S rRNA amplicon sequencing	✓	✓	✓	✓
	Metagenome	✓	✓	-	-

ASAQ, Adolescent Sedentary Activity Questionnaire; DXA, Dual-energy x-ray absorptiometry; EPOCH, Engagement Perseverance Optimism Connectedness Happiness; HbA1c, glycated haemoglobin; HOMA-IR, homeostasis model assessment of insulin resistance; hsCRP, high-sensitivity C-Reactive Protein; IBS, irritable bowel syndrome; IPAQ, International Physical Activity Questionnaire; NZAFFQ, New Zealand Adolescent food frequency questionnaire; PedsQL, Pediatric Quality of Life Inventory.

3.3 Withdrawals

Any participant who withdraws from the trial will be contacted by the clinical team. Attempts will be made to offer the participants the chance to provide crucial clinical information via follow up text messages or emails. All reasons for withdrawal will be recorded in secure databases, and will also be presented to the safety monitoring committee.

3.4 Research forms and data collection tools

All clinical assessments will be performed in accordance with Standard Operating Procedures and documented in relevant worksheets (i.e. case report forms – CRF) (Table 4).

Table 4. List of available case report forms (CRF) to be used in the Gut Bugs Trial.

**Demography**	Participant's questionnaire
	Caregiver's questionnaire
**Clinical history & physical examination [1]**	Recipient Worksheet – Baseline
	Recipient Worksheet – Week 6
	Recipient Worksheet – Week 12
	Recipient Worksheet – Week 26
**24-hour ambulatory BP monitoring**	24hr ABP worksheet
	Blood pressure report generated from Spacelabs Healthcare
	Sleep-activity diary
**DXA**	Report generated from Lunar Prodigy
	Report generated from Lunar iDXA
**Health-related quality of life**	EPOCH measure of adolescent well-being
	Paediatric Quality of Life Inventory (PedsQL)
	Birmingham symptom questionnaire
	Bowel movement questionnaire
**Diet and physical activity**	International physical activity questionnaire (IPAQ)
	Adolescent sedentary activity questionnaire (ASAQ)
	New Zealand Adolescent food frequency questionnaire (NZAFFQ)
	3-day food diary

[1] Includes records adverse events (if any) and blinding questions.

These are checked for completeness of data by the clinical research team. All questionnaires are checked for completeness. All paper CRFs, reports, questionnaires will be scanned and saved to the Gut Bugs Trial shared drive in appropriately named folders.

All questionnaires (except dietary ones) will be scored using specifically designed Microsoft Excel Spreadsheets for each questionnaire. Data will be entered once by the data entry person and validated by another data entry person. A final check will be carried out by the clinical research fellow.

All clinical data are to be entered into REDCap in accordance with the Data Management Standard Operating Procedure. Data will be entered once by the data entry person, validated by another data entry person, and finally checked and locked by the clinical research fellow. Below are the step-by-step process for data checking, which will be reflected by the REDCap Status stated below:

Incomplete Data entry is in progress and has been entered by first data entry person. Any missing information should be highlighted and efforts to obtain that information will be done by the clinical research team.

Unverified Data entry has been checked by second data entry person who will change the status from Incomplete to Unverified.

Complete All available data has been entered and verified.
Data is ready for cleaning and monitoring.
The data monitoring team will change status to Complete.

All complete data will be regularly checked by the trial statistician to identify potential data entry errors based on clinical plausibility. Dubious data points will be followed up and the respective clinical records checked.

Data that are to be imported into REDCap include:

1. NZIMD values
2. Paper questionnaires scores
3. BMI SDS values
4. Blood lipids
5. Liver function tests
6. Glucose, insulin, insulin sensitivity index values
7. Inflammatory markers

All data to be imported will be checked and cleaned by the trial statistician prior to importing into REDCap. Each variable entered into REDCap will be given a unique name and the completed codebook will be attached as an appendix to this document. Further details of data management are included in the Appendix 1 under Data Management Standard Operating Procedure.

3.5 Safety monitoring and evaluation

An independent safety monitoring committee has been established to for the duration of the trial. Participants' data will be monitored by the research team and the safety committee throughout the study for any adverse events, in particular gastrointestinal symptoms and possible allergies. Any possible adverse events are asked and recorded at 24 hours, 48 hours, 1 week, 3 weeks, 6 weeks, 12 weeks, and 26 weeks after the intervention in the adverse events worksheets. All potential adverse events will be recorded in a secure database. Any serious adverse event that is identified will be flagged and highlighted as soon as possible to the committee. If any participant suffers harm as a result of trial participation, they will be eligible to apply for compensation from the Accident Compensation Cooperation (ACC), which is a compulsory insurance cover for personal injury for everyone in New Zealand

3.6 Outcome variables and definitions

The study's outcome variables are listed in Table 5.

Table 5. List of key outcome variables in the Gut Bugs Trial.

Anthropometry	BMI SDS
Body composition (DXA)	Total body fat percentage, A/G ratio
Glucose metabolism	Matsuda index, HOMA-IR
Liver function	ALP, ALT, AST, GGT
Lipid profile	Total cholesterol, HDL, LDL, TG, total cholesterol/HDL, TG/HDL
Inflammatory markers	hsCRP, uric acid
Blood pressure	SBP, DPB, MAP, systolic dip, diastolic dip
Health-related quality of life	EPOCH scores, Peds QL scores, IBS symptoms scores, Bowel movements scores
Gut microbial composition	Alpha- and beta-diversities, relative abundance of bacterial taxa, donor strain engraftment

3.6.1 Body mass index standard deviation score (BMI SDS)

There will be triplicate measurements of weight and height. Median weight and height will be obtained and used to calculate BMI. The formula is BMI = kg/m^2 where kg is a person's weight in kilograms and m^2 is their height in metres squared. Subsequently, BMI SDS will be obtained as per World Health Organization standards [12].

3.6.2 Total body fat percentage (from DXA)

Total body fat percentage and A/G ratios will be obtained from DXA scan reports performed on the participants at these time points. The android region is defined as the area between the ribs and the pelvis that is totally enclosed by the trunk region. The gynoid region includes the hips and upper thighs and overlaps both the leg and trunk regions [13]. A/G ratio is calculated with the formula android fat mass/ gynoid fat mass [13]. As we will be utilising 2 different types of DXA machines (DXA, Lunar ProdigyTM and Lunar iDXA TM, GE Medical Systems, Chicago, Illinois, USA), all participants will have scans done at every time point on the same device.

3.6.3 Insulin sensitivity

Insulin sensitivity will be assessed in all recipients using the Matsuda index from a 75-g oral glucose tolerance test (OGTT)[14]. Blood samples will be collected at -10, 0, 30, 60, 90, and 120 minutes for glucose (mmol/L) and insulin (uU/ml) measurements. The insulin and glucose values will be used to obtain the Matsuda index [15]. The formula is 1000/square root of [fasting glucose x fasting insulin] x [mean glucose x mean insulin during OGTT] [15]. Other markers of glycaemic control will also be measured, namely homeostasis model assessment of insulin resistance (HOMA-IR)[16] and glycated haemoglobin (HbA1c) (mmol/mol). The formula for HOMA-IR is fasting insulin (uU/ml) x fasting glucose (mmol/L)/22.5. The normal reference ranges for glucose and insulin are provided in Table 6.

3.6.4 Liver function

Liver function will be assessed by measurement of gamma-glutamyl transferase (GGT) (U/L), alkaline phosphatase (ALP) (U/L), alanine aminotransferase (ALT) (U/L), and aspartate transaminase (AST) (U/L). The normal reference ranges for GGT, ALP, ALT and AST are provided in Table 6.

3.6.5 Lipid profile

Lipid profiles for the recipients will be assessed via measurement of fasting total cholesterol (mmol/L), high-density lipoprotein cholesterol [HDL] (mmol/L), low-density lipoprotein cholesterol [LDL] (mmol/L), and triglycerides (mmol/L). The normal reference ranges for total cholesterol, HDL, LDL, and triglycerides are provided in Table 6.

3.6.6 Inflammatory markers

Markers of inflammation will be assessed via uric acid (umol/L) and high-sensitivity C-reactive protein (hsCRP) (mg/L). The normal reference ranges for uric acid and hsCRP are provided in Table 6.

3.6.7 Blood pressure

Clinic resting systolic and diastolic blood pressures will be measured at all assessments using the same oscillometric digital blood pressure monitor (ri-champion® N; Riester, Jungingen, Germany) with an appropriately-sized cuff on the extended non-dominant arm. All measurements will be recorded on each recipient while seated and after a 5-minute rest. Blood pressure will be measured three times, and the median value calculated. The normal blood pressure readings will be following the European Society of Hypertension guidelines for the management of high blood pressure in children and adolescents [17].

In addition, 24-hour ambulatory blood pressure monitoring will be performed at baseline and at 6 weeks, using an oscillometric device (Spacelabs OnTrak; Spacelabs Medical Inc, Redmond, Washington, USA) on the non-dominant arm. Over a 24-hour period, blood pressure will be measured every 20 minutes when the recipients are expected to be awake, and every 30 minutes when they are likely to be asleep (based on self-reported information). Recipients will be asked to record the time they go to bed and the time they wake up over the period of monitoring, so that waking and sleeping times can be more accurately identified. The average overall blood pressure, awake blood pressure and the sleep blood pressure readings will be obtained. An adequate blood pressure report needs at least 14 day readings and 7 night readings [18]. The normal blood pressure readings will be following the European Society of Hypertension guidelines for the management of high blood pressure in children and adolescents [17].

3.6.8 Definitions of abnormal outcomes

Table 6 – Definitions of abnormal outcomes

OUTCOME	PARAMETER	THRESHOLDS FOR ABNORMAL RESULTS	REFERENCE
**Waist circumference**		14 years: ≥90th percentile (≥79.9 cm for males; ≥77 cm for females)	Zimmet et al. 2007[19]; Eisenmann et al. 2005[20]
		15 years: ≥90th percentile (≥81.7 cm for males; ≥78.4 cm for females)	
		≥16 years: ≥94 cm for males and ≥80 cm for females	
**Glucose homeostasis**	Elevated fasting glucose	≥5.6 but <7.0 mmol/L (impaired fasting glycaemia); ≥7.0 mmol/L(diabetes)	American Diabetes Association 2018[21]
	Elevated 1-hour glucose	≥8.6 mmol/mol	Fiorentino et al. 2018[22]
	Elevated 2-hour glucose	≥7.8 but <11.1 mmol/L (impaired glucose tolerance); ≥11.1 mmol/mol (diabetes)	American Diabetes Association 2018[21]
	Elevated HbA1c	≥39 to 47.9 mmol/mol (prediabetes); ≥48 mmol/mol (diabetes)	American Diabetes Association 2018[21]
		≥5.7 to 6.49 % (prediabetes); ≥6.5 % (diabetes)	
	Elevated fasting insulin	<15 years: >11.4 uU/ml for males and >14.0 uU/ml for females<br>≥15 years: >11.4 uU/ml for males and >12.9 uU/ml for females	Frithioff-Bøjsøe et al. 2019[23]
**Blood pressure**	Non-dipping status	Nocturnal drop in SBP and/or DBP ≤10%	Lurbe et al. 2016[17]
**Pre-hypertension**	24-hour ambulatory BP	SBP and/or DBP ≥90th but <95th percentile for age and sex	
	Clinic BP	<16 years: SBP and/or DBP ≥90th but <95th percentile for age and sex	
		≥16 years (high normal): SBP ≥130 but <140 mmHg and/or DBP ≥85 but <90 mmHg	
**Hypertension**	24-hour ambulatory BP	SPB and/or DBP ≥95th percentile for sex, age, and height, unless BP is equal to or higher than adult criteria thresholds (i.e. mean 24hr 130/80 mmHg; awake 135/85 mmHg; and sleep 125/75 mmHg)	
	Clinic BP	<16 years: SBP and/or DBP ≥95th percentile for age and sex	
		≥16 years: SBP and/or DBP ≥140/90 mmHg	
**Dyslipidaemia** [1]	HDL	<16 years: <1.03 mmol/L	Zimmet et al. 2007[19]
		≥16 years: males <1.03 mmol/L; females <1.29 mmol/L	
	LDL	>2.6 mmol/L	NCEP 2001[24]

OUTCOME	PARAMETER	THRESHOLDS FOR ABNORMAL RESULTS	REFERENCE
	Triglycerides	≥1.7 mmol/L	Zimmet et al. 2007[19]
	Total cholesterol	>5.2 mmol/L	European Atherosclerosis Society 1987[25]
**Hyperuricaemia**	Uric acid	Males ≥417 umol/L; females ≥340 umol/L	Thefeld et al. 1973[26]
**Elevated CRP**	hsCRP	<16 years: >2.8 mg/L	Schlebusch et al. 2002[27]
		≥16 years ≥5.0 mg/L	Dati et al. 1996[28]
**Abnormal liver function** [1]	ALP	<15 years: males >468 U/L; females >254 U/L	Estey et al. 2013[29]
		≥15 but <17 years: males >331 U/L; females >117 U/L	
		≥17 years: males >149 U/L; females >87 U/L	
	ALT	Males >41 U/L; females >33 U/L	Klein et al. 1994[30]
	AST	Males >40 U/L, females >32 U/L	Thefeld et al. 1974[31]
	GGT	Males ≥60 U/L, females ≥40 U/L	Thomas et al. 2005[32]
**Metabolic syndrome**		≥10 but <16 years:<br>Waist circumference ≥90th percentile (or adult cut-off if the latter is lower); AND any 2 of the following 4 criteria:<br>1. triglycerides ≥1.7 mmol/L<br>2. HDL <1.03 mmol/L<br>3. SBP ≥130 and/or DBP ≥85 mmHg<br>4. Fasting glucose ≥5.6 mmol/L and/or previously diagnosed type 2 diabetes<br><br>≥16 years:<br>Waist circumference ≥94 cm for males and ≥80 cm for females; AND any 2 of the following 4 criteria:<br>1. triglycerides ≥1.7mmol/L<br>2. HDL <1.03 mmol/L in males and <1.29 mmol/L in females; or specific treatment for these lipid abnormalities<br>3. SBP ≥130 mmHg and/or DBP ≥85 mmHg, or treatment for previously diagnosed hypertension<br>4. Fasting glucose ≥5.6 mmol/L and/or previously diagnosed type 2 diabetes	Zimmet et al. 2007[19]

All blood samples were analysed using the Roche/Hitachi cobas c 311 systems, except insulin, which was measured using Elecsys and cobas e 411 immunoassay analyzers.

[1] Adverse outcome observed when any of the listed parameters are abnormal.

3.6.9 EPOCH Measure of Adolescent Well-Being

This questionnaire provides an assessment of five positive psychological characteristics (engagement, perseverance, optimism, connectedness, and happiness) [33]. For each characteristic, there are 4 items. Each item is scored on a 1 to 5 scale (almost never/not at all like me=1; almost always/very much like me=5). Scores are computed for each characteristic as the average of the four items. If the score for any item is missing, the average for each characteristic would be calculated using the total score of items answered divided by the number of items answered.

3.6.10 Pediatric Quality of Life Inventory (PedsQL)

We will adopt only the teen and young adult self-reports (*i.e.* not the parent-proxy), which assess problems over the preceding month relating to physical, emotional, social, and school functioning [34]. This questionnaire consists of 23 items comprising of 4 sections (physical functioning, emotional functioning, social functioning and school functioning). Each item is scored on a 0 to 4 scale (0= never; 4= almost always). Each score will then be transformed on a scale from 0 to 100 (0=100, 1=75, 2=50, 3=25, 4=0). If more than 50% of the items in the scale are missing, the scale scores should not be computed. Mean scores are the sum of the items over the number of items answered. We will also obtain the psychosocial health summary score and total score from the 4 sections. The psychosocial health summary score is sum of the items over the number of items answered in the emotional, social and school functioning sections. The total score is the sum of all items over the number of items answered on all sections.

3.6.11 IBS symptoms

The Birmingham IBS symptom questionnaire is a self-administered 11-item symptom questionnaire that is scored using the Rome II criteria [35]. There are 3 sections; pain, constipation and diarrhea. Each item is scored on a scale from 0 to 5 (0= none of the time; 5= all of the time). The total score is a sum of all 3 sections. If more than 50% of the items are missing, the scores should not be computed.

3.6.12 Bowel movements

The bowel movement questionnaire was designed for this trial to assess and monitor changes pre and post treatment. There are 5 items in the questionnaire. Item 1, 3, 4 and 5 are scored on a scale of 1 to 5. Item 2 is scored on a scale from 1 to 7. A higher score indicates a reduction in

bowel movement. If more than 50% of the items are missing, the scores should not be computed.

3.6.13 Gut microbial composition

Sample collection will be performed at baseline prior to treatment and at 6 weeks, 12 weeks and 26 weeks post-treatment. Briefly, the participant will be given the bedpan liner (Onelink). They will be asked to:
i) pass urine into the toilet prior to placing the tray on the toilet seat;
ii) pass the stools;
iii) cover the tray and leave it in the bathroom for immediate collection by a research team member.
Using a small spatula, samples will be collected from three different areas of the stool (proximal, middle, and distal) and inserted into specimen containers (Onelink). The specimen containers will be immediately placed on ice and taken to the laboratory where they will be frozen and stored at -80°C. DNA and RNA extraction will be completed within 5 days of donation. Time to processing will be recorded.

Note that we will advise participants to try not to have a bowel movement in the morning prior to their visit, having it in the clinic instead. For those participants who are unable to produce a stool sample during their visit, they will be provided with a stool collection kit to take home and detailed instructions on how to collect the stool sample. This kit is made up of: i) instructions on how to use the stool collection kit; ii) specimen container; and iii) bedpan liner. Once the stool has been collected in the home environment, the specimen container it should be immediately placed into their home freezer, and kept there until it is delivered to the research team.

All extractions will be performed using Qiagen-AllPrep DNA/RNA mini kit®, due to variation in extraction efficiencies with the different kits [36]. However, once the DNA or RNA is extracted and archived, we will have a relatively stable record of the composition and activity of the flora.

Frozen faeces (~200 mg; weights will be recorded) will be subsampled from original faecal samples. All DNA and RNA isolations will be performed in a disinfected class II hood at room temperature. Briefly, stool samples will be incubated (10 min, room temperature) with vortexing (30 sec every 2 minutes) and treated with RLT Plus buffer (1.2mL; Qiagen) and 12µL beta-mercaptoethanol (Sigma-Aldrich). Acid-washed glass beads [1 ml; ≤106 µm (-140 U.S. sieve) (Sigma-Aldrich)] will be added to each sample and vortexed (10 min) on a TissueLyzer II (Qiagen). The supernatant will be removed and added to a QIAshredder spin column (Qiagen)

and centrifuged (9000 rpm, 2 min, room temperature). The eluent will be added to an AllPrep DNA (Qiagen) spin column and centrifuged (30 sec, 14000 rpm, room temperature). The eluent and AllPrep DNA spin columns will be used for RNA and DNA extraction, respectively, according to the manufacturer's instructions. Finally, DNA and RNA will be eluted with EB buffer and RNase-free water, respectively, and aliquots stored at -80°C for downstream mixed omics analysis.

A series of blank samples (sterile saline) will be extracted in parallel to sample extractions to enable contamination testing. We will also extract ZymoBIOMICS™ Microbial Community Standard I (Even, Cellular Mix; Catalog #D6300) to determine potential bias in the extraction process.

For 16S amplicon sequencing, library preparation will be performed using an Illumina platform by a commercial provider (to be determined) using standard protocols for the SV3-4 region. Shotgun metagenomics sequencing will be performed by a commercial provider (to be determined).

3.7 Diet, physical activity, and socioeconomic status

We will also be collecting data throughout the trial on lifestyle parameters that could possibly affect treatment outcomes, namely physical activity levels, dietary intake and socioeconomic status. Physical activity levels will be assessed via the IPAQ and ASAQ scores and the dietary intake will be assessed via the NZAFFQ questionnaire.

3.7.1 IPAQ

The IPAQ assesses physical activity for 4 domains including work-related physical activity, transport-related physical activity, domestic and gardening activities and leisure time physical activity [37,38]. The items in the IPAQ are structured to provide separate domain specific scores for walking, moderate-intensity, and vigorous-intensity activity. Computation of the total scores requires summation of the duration (in minutes) and frequency (days) for all the types of activities in all domains. Domain specific scores require summation of the scores for walking, moderate-intensity, and vigorous-intensity activities (work-related physical activity, transport-related physical activity, domestic and gardening activities and leisure time physical activity). Total score will be a sum of all the domain specific scores. Activity-specific scores require summation of all the scores for the specific type of activity across domains (total vigorous activity, total moderate activity, total moderate and vigorous activity). We can also generate a categorical score (low, moderate and high) for the different levels of physical activity. The

amount of time spent sitting can be obtained from the time spent sitting during the weekday and weekend.

• Total MET-minutes/week at work = Walk (METs*min*days) + Mod (METs*min*days) + Vig (METs*min*days) at work
• Total MET-minutes/week for transportation = Walk (METs*min*days) + Cycle (METs*min*days) for transportation
• Total MET-minutes/week from domestic and garden = Vig (METs*min*days) yard work + Mod (METs*min*days) yard work + Mod (METs*min*days) inside chores
• Total MET-minutes/week in leisure-time = Walk (METs*min*days) + Mod (METs*min*days) + Vig (METs*min*days) in leisure-time
• Total Walking MET-minutes/week = Walk MET-minutes/week (at Work + for Transport + in Leisure)
• Total Moderate MET-minutes/week = Cycle MET-minutes/week for Transport + Mod MET-minutes/week (Work + Yard chores + Inside chores + Leisure) + Vigorous Yard chores MET-minutes
• Total Vigorous MET-minutes/week = Vig MET-minutes/week (at Work + in Leisure)

IPAQ categorical scoring:

1. Low: no activity is reported; OR
some activity is reported but not enough to meet Categories 2 or 3.

2. Moderate: One of the following 3 criteria is met:
a) 3 or more days of vigorous-intensity activity of at least 20 minutes per day; OR
b) 5 or more days of moderate-intensity activity and/or walking of at least 30 minutes per day; OR
c) 5 or more days of any combination of walking, moderate-intensity or vigorous-intensity activities achieving a minimum of at least 600 MET-min/week.

3. High: One of the following 2 criteria is met:
a) vigorous-intensity activity on at least 3 days and accumulating at least 1500 MET-minutes/week; OR
b) 7 or more days of any combination of walking, moderate- or vigorous- intensity activities accumulating at least 3000 MET-minutes/week.

Further details of the IPAQ scoring is provided in the Guidelines for Data Processing and Analysis of the International Physical Activity Questionnaire (IPAQ) [38].

3.7.2 ASAQ

The ASAQ measures time spent in sedentary behaviours among adolescents [39]. There are 5 domains that are assessed which include, small screen recreation, education, travel, cultural activities and social activities. Time spent during weekday and weekend in each domain is recorded to obtain total weekday, total weekend and total week scores.

3.7.3 NZAFFQ

The New Zealand Adolescent Food Frequency Questionnaire (NZAFFQ) [40] provides the frequency of eating different types of food groups. This information can be used to compare frequency of food group intake between the two intervention groups and across the duration of the intervention.

3.7.4 3-day food diary

This diary will also be used to describe all foods and fluids consumed over three days. Recipients will be asked to describe all foods and fluids consumed in detail including brand names, types of foods (e.g. low fat), and cooking methods. Quantities will be described using standard household measures, as well as the information from food labels (where appropriate). Recipients will be provided with standardized instructions for completing the dietary record by a trained investigator, who will also review individual records with recipients to clarify errors, omissions, questionable entries, or unclear descriptions. These dietary records will be entered into FoodWorks software (v9.0, Xyris Software, Brisbane, Australia) by a trained investigator.

3.7.5 Socioeconomic status

This is evaluated via the New Zealand indices of multiple deprivation (IMD) scores [41]. There are seven domains of deprivation; employment, income, crime, housing, health, education; and geographical access. The overall IMD score is the combination of these seven domains. To generate this scores, the participant’s residential address is entered into qualtrics survey which will then convert the information provided into the scores.

4 STATISTICAL METHODS

The CONSORT 2010 guidelines will be followed in reporting the main trial results [1,2]. Data analyses will be performed in SAS v.9.4 (SAS Institute, Cary, NC, USA), SPSS v25 (IBM Corp, Armonk, NY, USA), and/or Minitab v.16 (Pennsylvania State University, State College, PA, USA). All statistical tests will be two-sided at $p<0.05$, with no adjustments for multiple comparisons. Baseline demographics and clinical characteristics of recipients will be summarised by randomisation group. The distribution of outcome measures will be first evaluated using descriptive statistics. No interim analysis is planned for the trial.

4.1 Primary outcome analyses

Treatment evaluation will be performed on the principle of intention to treat (ITT), using data collected from all randomised recipients.

A linear regression model will be used to assess the treatment effect between two groups on the primary outcome (BMI SDS) at 6 weeks, adjusting for the baseline outcome value and sex (i.e. stratification factor). Model-adjusted estimates and the differences between the two groups will be calculated with 95% confidence intervals.

Missing data on the primary outcome will be imputed using multiple imputations, which create multiple imputed datasets for the incomplete outcome variable that are analysed using same regression models and combined for one inference. The Markov chain Monte Carlo (MCMC) method will be used to produce the parameter estimates, assuming the data are from a multivariate normal distribution and are missing at random. The SAS procedure, PROC MI, will be used, and we plan to run 30 imputations to allow for both within and between imputation variances.

Per-protocol analyses may be carried out on those randomised participants without major protocol violations, which have been defined as any of the following:

• Extreme changes to dietary intake during the trial; ie starting on a new type of dieting programme

• Extreme changes to physical activity during the trial; ie starting a new type of high-intensity exercise

• diagnosed during the trial to have a medical condition listed under the exclusion criteria to be eligible for the trial (i.e. gastrointestinal disease, type 1 diabetes or monogenic diabetes that

requires being on medication that may potentially affect weight, metabolism or the gut microbiome)

• starting regular medications during the trial that may influence weight, metabolism or gut microbiome (i.e. oral contraceptives, metformin)

A protocol deviation form will be used to record all major protocol deviations, and reviewed in a blinded fashion by the trial steering group prior to final data lock. The per-protocol population will be analysed using same regression models as the primary ITT population to test the robustness of main trial findings.

Planned subgroup analysis by sex will be conducted to evaluate the consistency of main treatment effects in males and females, by including an interaction term between sex and treatment group in the main model. If a significant interaction effect is found, separate subgroup analyses will be conducted to estimate the treatment effects in specific subgroups.

Exploratory analyses may also be performed following the exact same procedures as previously described, accounting for the potential confounding effects of physical activity levels and dietary intake.

4.2 Secondary outcome analyses

Treatment evaluation will be performed on the principle of intention to treat (ITT), using data collected from all randomised recipients on previously specified secondary outcomes. Generalised linear mixed models will be used to evaluate the outcomes measured repeatedly over time, using a link function appropriate to the distribution of the outcome variable. The fixed-effect model will include the value of the respective outcome at baseline, sex (stratification factor), treatment group, visit, and the latter's interaction with treatment group. A random patient effect will be considered in modelling to take into account the correlation between the data collected from same participant. Model-adjusted group differences will be estimated at each clinical assessment with 95% confidence intervals. The interaction effect between treatment group and visit will be assessed.

5 MICROBIOME DATA ANALYSIS

5.1 Initial bioinformatics

The metagenomic sequencing data will be processed using bioBakery workflows using docker images available at http://huttenhower.sph.harvard.edu/biobakery_workflows. Briefly, sequence data quality control, including removal of any human reads, will be conducted using kneadData. Taxonomic and functional profiles of the microbiome will be generated using MetaPhlAn v2.6 [42] and HUMAnN2 [43], respectively. Additionally, strain level taxonomic profiling will be achieved by SNP haplotype based profiling by StrainPhlAn software [44]. Quality control, read filtering, trimming and dereplicatiion, read-pair joining, sequence denoising, chimera removal and sequence table construction of 16S rRNA amplicon sequencing data will be conducted using DADA2 R package [45]. Sequence taxonomies will be assigned using IDTAXA algorithm in DECIPHER R package [46].

5.2 Metagenomic assembly

Metaganomic reads of each sample will be assembled into contigs separately using MegaHIT [47]. Open reading frames (ORFs) of the assembled contigs will be predicted using Prodigal [48] followed up by clustering the ORFs with >95% identity and 90% coverage into non-redundant gene clusters by CD-HIT [49,50]. The resulting non-redundant gene catalogue will be merged using existing gene catalogues to assure more comprehensive reference gene catalogue [51,52]. Gene abundance will be determined by mapping reads from the metagenomic samples to the gene catalogue using Burrows-Wheeler Aligner (BWA) [53]. The resulting gene abundance profiles will be used to construct microbial pangenomes using MPSminer [54]. Similarity between a pair of metagenomic strains (within the same species but in different samples) will be measured using the percentage of shared genes in the smallest of the two genomes [55].

5.3 Statistical comparisons

The microbiomes of the treatment groups (FMT vs. placebo) will be compared using both omnibus (Permutational analysis of variance, PERMANOVA) and individual (linear mixed effects modeling) feature tests. Both taxonomic (based on 16S amplicon and metagenomic sequencing) and functional (metagenomic sequencing) profiles will be compared. Following comparisons for both sexes separately (males and females) and all data as an aggregate (but by controlling sex as a covariate) will be included:

1) Compare first post-treatment samples to look for consistent post-treatment differences between the groups.
2) Compare pre- and post-treatment alpha-diversities, and shifts in alpha-diversity between the groups.

3) Compare microbial stability (Bray-Curtis dissimilarity and change in any individual taxa) over the treatment between the groups.

4) Compare pre- and post-treatment similarities to donors (as an aggregate and individually) between groups.

5.4 Engraftment analysis

We will use the SNP haplotypes from StrainPhlAn [44] to investigate microbial engraftment at the strain level. We will first select a sequence similarity threshold for matching donor-recipient strains by using the placebo group as a negative control; no strain transfer (or engraftment) should be observed in placebo group. We will then treat all donor strains with higher than this previously selected sequence similarity in recipient stool as successfully engrafted strains. We will quantify engraftment per participant by counting the number of engrafted strains and by measuring their total abundance in the recipient gut microbiome. We will then correlate these engraftment measures to other clinical data, such as weight loss, quality of life and reported post-treatment adverse effects.

The metagenomic assemblies will be used to confirm the results of SNP haplotype based analysis above and to discover engraftment of genomes that are missing or not well-presented in the reference databases. As above, the gene content similarity threshold for matching donor-recipient strains will be determined experimentally using the placebo group as a negative control. The metagenomic assemblies will also be used to discover rare genes that are shared between donor-recipient pairs and which may participate in the trophic cascade following the FMT.

5.5 Identification of super-donor behavior

We will analyse the data to identify any evidence supporting the existence of super-donor(s) in this study using the following tests:

1) Measure recipient shifts (in Bray-Curtis dissimilarity and Jaccard Index) towards all donor microbiome profiles separately and compare these shifts using ANOVA.
2) Compare donors by counting the engrafted strains (and their relative abundance) as identified as above.
3) Assess any anecdotal trends and associations between donor alpha diversities and engraftment of their strains.

6 REPORTING OF TRIAL RESULTS

6.1 Baseline characteristics

Table 7. Baseline demographic and clinical characteristics of participants

		Placebo	Treatment
n			
Age (years)			
Sex (males)			
Socioeconomic deprivation (IMD)			
Ethnicity	NZ European		
	Maori		
	Pacific Islander		
	Other ethnicities		
Anthropometry	Height (cm)		
	Weight (kg)		
	Class 1 obesity (%)		
	Class 2 obesity (%)		
	Class 3 obesity (%)		
Body composition	Total body fat (%)		
Glucose homeostasis	Elevated fasting glucose (%)		
	Elevated insulin (%)		
Blood pressure	Pre-hypertension (%)		
	Hypertension (%)		
Lipid profile	Dyslipidaemia (%)		
Liver function	Abnormal (%)		

6.2 Study outcomes

Table 8. Primary outcome- BMI SDS at 6 weeks post-intervention. Data are means and the respective 95% confidence intervals.

		Treatment		Placebo		Adjusted difference (95% CI)
		Baseline (mean,SD)	6 weeks (mean,SD)	Baseline (mean,SD)	6 weeks (mean,SD)	
ITT	BMI SDS					
PP	BMI SDS					

Table 9. Clinical outcomes at baseline, 6, 12 and 26 weeks post-intervention. Data are means and the respective 95% confidence intervals.

		Baseline		6 weeks			12 weeks			26 weeks		
		Treatment	Placebo	Treatment	Placebo	Difference	Treatment	Placebo	Difference	Treatment	Placebo	Difference
Anthropometry	Waist circumference (cm)											
	Hip circumference (cm)											
Body composition	Total body fat (%)											
	Android: gynoid fat ratio											
Glucose homeostasis	Matsuda index											
	Fasting plasma glucose (mmol/L)											
	Fasting insulin (uU/ml)											
	HOMA-IR											
	HbA1c (mmol/mol)											
Clinic blood pressure	Systolic (mmHg)											
	Diastolic (mmHg)											
Lipid profile	Total cholesterol (mmol/L)											
	LDL (mmol/L)											
	HDL (mmol/L)											
	Triglycerides (mmol/L)											
	Total cholesterol / HDL											
	Triglycerides / HDL											
Inflammatory markers	Uric acid (umol/L)											
	hsCRP (mg/L)											
Liver function	ALP											
	ALT											
	AST											
	GGT											

Table 10. Health-related quality of life outcomes at Baseline, 6, 12 and 26 weeks post-intervention. Data are means and the respective 95% confidence intervals.

		Baseline		6 weeks			12 weeks			26 weeks		
		Treatment	Placebo	Treatment	Placebo	Difference	Treatment	Placebo	Difference	Treatment	Placebo	Difference
**EPOCH**	Engagement											
	Perseverance											
	Optimism											
	Connectedness											
	Happiness											
**PedsQL**	Physical functioning											
	Emotional functioning											
	Social functioning											
	School functioning											
	Psychosocial health											
	Total											
**IBS symptoms**	Pain											
	Constipation											
	Diarrhoea											
	Total											
**Bowel movements**	Total											

Table 11. Microbial alpha- and beta-diversities post-intervention. Data are means and the respective 95% confidence intervals.

		Placebo	Treatment	Difference	*p*
**n**					
**Alpha-diversity**	at 6 weeks				
	at 12 weeks				
	at 26 weeks				
	change compared to baseline at 6 weeks				
	change compared to baseline at 12 weeks				
	change compared to baseline at 26 weeks				
**Beta-diversity**	between baseline and 6 weeks				
	between baseline and 12 weeks				
	between baseline and 26 weeks				
	between donor stool				

Table 12. Associations between study variables and microbial taxa. Effect sizes are differences of means between the groups or correlation coefficients.

Study variable	Bacterial taxon	Effect size	n	*p*	FDR corrected *p*

Table 13. Outcome variables for Gut Bugs Trial's main manuscript

*Indicates categorical (binary) outcomes, as defined in Table 6

[ ] Analysis of potential treatment effects on individual components may not be carried out in the absence of overall differences.

		OUTCOMES	TIME POINTS
**PRIMARY OUTCOME**			
**Anthropometry**		BMI SDS	6 weeks
**SECONDARY OUTCOMES**			
**Anthropometry**		BMI SDS	12, 26 weeks
		Waist circumference (cm)	6, 12, 26 weeks
		Waist-to-height ratio	6, 12, 26 weeks
**Body composition (DXA)**		Total body fat (%)	6, 12, 26 weeks
		Android-to-gynoid fat ratio	6, 12, 26 weeks
		Total lean mass (kg)	6, 12, 26 weeks
**Blood pressure**	**Clinic**	Median systolic BP (mmHg)	6, 12, 26 weeks
		Median diastolic BP (mmHg)	6, 12, 26 weeks
		*Pre-hypertension	6, 12, 26 weeks
		*Hypertension	6, 12, 26 weeks
	**24-hour**	Awake mean systolic BP (mmHg)	6 weeks
		Awake mean diastolic BP (mmHg)	6 weeks
		Sleep mean systolic BP (mmHg)	6 weeks
		Sleep mean diastolic BP (mmHg)	6 weeks
		Systolic dip (%)	6 weeks
		Diastolic dip (%)	6 weeks
		*Pre-hypertension	6 weeks
		*Hypertension	6 weeks
**Metabolism**	**Glucose homeostasis**	Insulin sensitivity (Matsuda Index)	6, 12, 26 weeks
		Fasting insulin (uU/ml)	6, 12, 26 weeks
		Fasting glucose (mmol/L)	6, 12, 26 weeks
		HbA1c (mmol/mol)	6, 12, 26 weeks
		HOMA-IR	6, 12, 26 weeks
		*Elevated fasting glucose	6, 12, 26 weeks

		OUTCOMES	TIME POINTS
		*Elevated 1-hour glucose	6, 12, 26 weeks
		*Elevated 2-hour glucose	6, 12, 26 weeks
		*Pre-diabetes or diabetes on HbA1c	6, 12, 26 weeks
	**Liver function**	ALP (U/L)	6, 12, 26 weeks
		ALT (U/L)	6, 12, 26 weeks
		AST (U/L)	6, 12, 26 weeks
		GGT (U/L)	6, 12, 26 weeks
		*Abnormal liver function	6, 12, 26 weeks
	**Lipid profile**	Total cholesterol (mmol/L)	6, 12, 26 weeks
		HDL (mmol/L)	6, 12, 26 weeks
		Triglycerides (mmol/L)	6, 12, 26 weeks
		LDL (mmol/L)	6, 12, 26 weeks
		Triglycerides/HDL	6, 12, 26 weeks
		*Dyslipidaemia	6, 12, 26 weeks
	**Inflammatory markers**	High-sensitivity CRP (mg/L)	6, 12, 26 weeks
		Uric acid (mmol/L)	6, 12, 26 weeks
	***Metabolic syndrome**		6, 12, 26 weeks
**Health-related quality of life**	**EPOCH**	[Engagement]	[6, 12, 26 weeks]
		[Perseverance]	[6, 12, 26 weeks]
		[Optimism]	[6, 12, 26 weeks]
		[Connectedness]	[6, 12, 26 weeks]
		[Happiness]	[6, 12, 26 weeks]
	**Peds QL**	Total score	6, 12, 26 weeks
		[Physical health]	[6, 12, 26 weeks]
		[Emotional]	[6, 12, 26 weeks]
		[Social]	[6, 12, 26 weeks]
		[School]	[6, 12, 26 weeks]
		[Psychosocial]	[6, 12, 26 weeks]

		OUTCOMES	TIME POINTS
**Gut health**	**IBS symptoms**	Total score	6, 12, 26 weeks
		Pain	6, 12, 26 weeks
		Constipation	6, 12, 26 weeks
		Diarrhoea	6, 12, 26 weeks
	**Bowel movements**	Total score	6, 12, 26 weeks

7 REFERENCES

1. Moher D, Hopewell S, Schulz KF, et al. CONSORT 2010 explanation and elaboration: updated guidelines for reporting parallel group randomised trials. *Int J Surg.* 2012;10(1):28-55.
2. Schulz KF, Altman DG, Moher D. CONSORT 2010 statement: updated guidelines for reporting parallel group randomised trials. *BMC Med.* 2010;8(1):18.
3. Chan AW, Tetzlaff JM, Altman DG, et al. SPIRIT 2013 statement: defining standard protocol items for clinical trials. *Ann Intern Med.* 2013;158(3):200-207.
4. New Zealand Blood Service. Detailed eligibility criteria. 2015.
5. Bibby K, Peccia J. Identification of viral pathogen diversity in sewage sludge by metagenome analysis. *Environ Sci Technol.* 2013;47(4):1945-1951.
6. Lacy BE, Patel NK. Rome criteria and a diagnostic approach to irritable bowel syndrome. *Journal of clinical medicine.* 2017;6(11).
7. Vanner SJ, Depew WT, Paterson WG, et al. Predictive value of the Rome criteria for diagnosing the irritable bowel syndrome. *Am J Gastroenterol.* 1999;94(10):2912-2917.
8. Sealed Envelope Ltd. Create a blocked randomisation list. 2016; https://www.sealedenvelope.com/simple-randomiser/v1/lists. Accessed 3 Mar 2017.
9. Bang H, Ni L, Davis CE. Assessment of blinding in clinical trials. *Control Clin Trials.* 2004;25(2):143-156.
10. Moroz A, Freed B, Tiedemann L, Bang H, Howell M, Park JJ. Blinding measured: a systematic review of randomized controlled trials of acupuncture. *Evid Based Complement Alternat Med.* 2013;2013(708251):12.
11. O'Brien PE, Sawyer SM, Laurie C, et al. Laparoscopic adjustable gastric banding in severely obese adolescents: a randomized trial. *JAMA.* 2010;303(6):519-526.
12. de Onis M, Blossner M, Borghi E. Global prevalence and trends of overweight and obesity among preschool children. *Am J Clin Nutr.* 2010;92(5):1257-1264.
13. Imboden MT, Welch WA, Swartz AM, et al. Reference standards for body fat measures using GE dual energy x-ray absorptiometry in Caucasian adults. *PLoS One.* 2017;12(4):e0175110-e0175110.
14. Matsuda M, DeFronzo RA. Insulin sensitivity indices obtained from oral glucose tolerance testing: comparison with the euglycemic insulin clamp. *Diabetes Care.* 1999;22(9):1462-1470.
15. Matsuda M, DeFronzo R. Insulin sensitivity indices obtained from oral glucose tolerance testing: comparison with the euglycemic insulin clamp. *Diabetes Care.* 1999;22(9):1462-1470.
16. Wallace TM, Levy JC, Matthews DR. Use and abuse of HOMA modeling. *Diabetes Care.* 2004;27(6):1487-1495.
17. Lurbe E, Agabiti-Rosei E, Cruickshank JK, et al. 2016 European Society of Hypertension guidelines for the management of high blood pressure in children and adolescents. *J Hypertens.* 2016;34(10):1887-1920.
18. O'brien E, Asmar R, Beilin L, et al. European Society of Hypertension recommendations for conventional, ambulatory and home blood pressure measurement. *J Hypertens.* 2003;21(5):821-848.
19. Zimmet P, Alberti KGM, Kaufman F, et al. The metabolic syndrome in children and adolescents–an IDF consensus report. *Pediatr Diabetes.* 2007;8(5):299-306.
20. Eisenmann JC. Waist circumference percentiles for 7- to 15-year-old Australian children. *Acta Paediatr.* 2005;94(9):1182-1185.
21. American Diabetes Association. Classification and Diagnosis of Diabetes: Standards of Medical Care in Diabetes 2018. *Diabetes Care.* 2018;41(Supplement 1):S13-S27.
22. Fiorentino TV, Marini MA, Succurro E, et al. One-hour postload hyperglycemia: implications for prediction and prevention of type 2 diabetes. *J Clin Endocrinol Metab.* 2018;103(9):3131-3143.
23. Frithioff-Bøjsøe C, Lund MA, Kloppenborg JT, et al. Glucose metabolism in children and adolescents: population-based reference values and comparisons to children and adolescents enrolled in obesity treatment. *Pediatr Diabetes.* 2019;20(5):538-548.
24. NCEP Expert Panel. Executive Summary of The Third Report of The National Cholesterol Education Program (NCEP) Expert Panel on Detection, Evaluation, And Treatment of High Blood Cholesterol In Adults (Adult Treatment Panel III). *JAMA.* 2001;285(19):2486-2497.
25. European Atherosclerosis Society. Strategies for the prevention of coronary heart disease: a policy statement of the European Atherosclerosis Society. *Eur Heart J.* 1987;8(1):77-88.
26. Thefeld W, Hoffmeister H, Busch E-W, Koller P, Vollmar J. [Normal values of serum uric acid depending on age and sex with one new enzymatic uric acid color test]. *Dtsch Med Woschenschr.* 1973;98(8):380-384.
27. Schlebusch H, Liappis N, Kalina E, Klein C. High sensitive CRP and creatinine: reference intervals from infancy to childhood. *J Lab Med.* 2002;26(5-6):341-346.
28. Dati F, Schumann G, Thomas L, et al. Consensus of a group of professional societies and diagnostic companies on guidelines for interim reference ranges for 14 proteins in serum based on the standardization against the IFCC/BCR/CAP reference material (CRM 470). *Eur J Clin Chem Clin Biochem.* 1996;34(6):517-520.
29. Estey MP, Cohen AH, Colantonio DA, et al. CLSI-based transference of the CALIPER database of pediatric reference intervals from Abbott to Beckman, Ortho, Roche and Siemens Clinical Chemistry Assays: direct validation using reference samples from the CALIPER cohort. *Clin Biochem.* 2013;46(13-14):1197-1219.
30. Klein G, Lehmann P, Michel E, Regenauer H. [Comparison of the IFCC methods for ALT, AST and GGT at 37°C with the established standard methods at 25°C and 37°C]. *Lab Med.* 1994;18:403-404.
31. Thefeld W, Hoffmeister H, Busch E-W, Koller P, Vollmar J. [Reference values for the determinations of the transaminases GOT and GPT as well as serum alkaline phosphatase using optimized standard methods]. *Dtsch Med Wochenschr.* 1974;99(8):343-351.
32. Thomas L, Müller M, Schumann G, et al. Consensus of DGKL and VDGH for interim reference intervals on enzymes in serum *J Lab Med.* 2005;29(5):301-308.
33. Kern ML, Benson L, Steinberg EA, Steinberg L. The EPOCH Measure of Adolescent Well-Being. *Psychol Assess.* 2016;28(5):586-597.
34. Varni JW, Seid M, Knight TS, Uzark K, Szer IS. The PedsQL 4.0 Generic Core Scales: sensitivity, responsiveness, and impact on clinical decision-making. *J Behav Med.* 2002;25(2):175-193.
35. Roalfe AK, Roberts LM, Wilson S. Evaluation of the Birmingham IBS symptom questionnaire. *BMC Gastroenterol.* 2008;8:30.

36. Claassen S, du Toit E, Kaba M, Moodley C, Zar HJ, Nicol MP. A comparison of the efficiency of five different commercial DNA extraction kits for extraction of DNA from faecal samples. *J Microbiol Methods.* 2013;94(2):103-110.
37. Hagströmer M, Oja P, Sjöström M. The International Physical Activity Questionnaire (IPAQ): a study of concurrent and construct validity. *Public Health Nutr.* 2006;9(6):755-762.
38. IPAQ. Guidelines for data processing and analysis of the International Physical Activity Questionnaire (IPAQ)-short and long forms. 2005.
39. Hardy LL, Booth ML, Okely AD. The reliability of the adolescent sedentary activity questionnaire (ASAQ). *Prev Med.* 2007;45(1):71-74.
40. Wong JE, Parnell WR, Black KE, Skidmore PM. Reliability and relative validity of a food frequency questionnaire to assess food group intakes in New Zealand adolescents. *Nutr J.* 2012;11(1):65.
41. Exeter D, Browne MM, Crengle S, Lee A, Zhao J. New Zealand Indices of Multiple Deprivation (IMD). *PLoS One.* 2016;12(8):e0181260.
42. Segata N, Waldron L, Ballarini A, Narasimhan V, Jousson O, Huttenhower C. Metagenomic microbial community profiling using unique clade-specific marker genes. *Nat Methods.* 2012;9(8):811-814.
43. Franzosa EA, McIver LJ, Rahnavard G, et al. Species-level functional profiling of metagenomes and metatranscriptomes. *Nat Methods.* 2018;15:962–968.
44. Truong DT, Tett A, Pasolli E, Huttenhower C, Segata N. Microbial strain-level population structure and genetic diversity from metagenomes. *Genome Res.* 2017;27(4):626-638.
45. Callahan BJ, McMurdie PJ, Rosen MJ, Han AW, Johnson AJ, Holmes SP. DADA2: High-resolution sample inference from Illumina amplicon data. *Nat Methods.* 2016;13(7):581-583.
46. Murali A, Bhargava A, Wright ES. IDTAXA: a novel approach for accurate taxonomic classification of microbiome sequences. *Microbiome.* 2018;6(1):140.
47. Li D, Liu CM, Luo R, Sadakane K, Lam TW. MEGAHIT: an ultra-fast single-node solution for large and complex metagenomics assembly via succinct de Bruijn graph. *Bioinformatics.* 2015;31(10):1674-1676.
48. Hyatt D, Chen GL, Locascio PF, Land ML, Larimer FW, Hauser LJ. Prodigal: prokaryotic gene recognition and translation initiation site identification. *BMC Bioinformatics.* 2010;11:119.
49. Qin J, Li R, Raes J, et al. A human gut microbial gene catalogue established by metagenomic sequencing. *Nature.* 2010;464(7285):59-65.
50. Fu L, Niu B, Zhu Z, Wu S, Li W. CD-HIT: accelerated for clustering the next-generation sequencing data. *Bioinformatics.* 2012;28(23):3150-3152.
51. Li J, Jia H, Cai X, et al. An integrated catalog of reference genes in the human gut microbiome. *Nat Biotechnol.* 2014;32(8):834-841.
52. Vatanen T, Plichta DR, Somani J, et al. Genomic variation and strain-specific functional adaptation in the human gut microbiome during early life. *Nat Microbiol.* 2018.
53. Li H, Durbin R. Fast and accurate short read alignment with Burrows-Wheeler transform. *Bioinformatics.* 2009;25(14):1754-1760.
54. Plaza Onate F, Le Chatelier E, Almeida M, et al. MSPminer: abundance-based reconstitution of microbial pan-genomes from shotgun metagenomic data. *Bioinformatics.* 2018.
55. Snel B, Bork P, Huynen MA. Genome phylogeny based on gene content. *Nat Genet.* 1999;21(1):108-110.
