# Supplementary material for: Effects of Fecal Microbiome Transfer in Adolescents With Obesity: The Gut Bugs Randomized Controlled Trial
Source: JAMA Netw Open. 2020 Dec 21;3(12):e2030415. doi: 10.1001/jamanetworkopen.2020.30415 (PMC7753902; doi:10.1001/jamanetworkopen.2020.30415)
Supplement: Supplement 3. — eTable 1. Android-to-Gynoid-Fat (A/G) Ratio at 6, 12, and 26 Weeks Postintervention Among Female and Male Adolescents eTable 2. Secondary Outcomes at 6, 12, and 26 Weeks Postintervention eTable 3. BMI SDS and Glucose Metabolism of Participants with Metabolic Syndrome at Baseline eTable 4. Changes in Prevalence of Metabolic Syndrome Among Participants with Metabolic Syndrome at Baseline eTable 5. Variance of Species Taxonomic Profiles (Bray-Curtis Dissimilarity) Explained by Each Covariate in Cross-sectional PERMANOVA eTable 6. Variance of Species Taxonomic Profiles (Bray-Curtis dissimilarity) Explained by Each Covariate in PERMANOVA for Donors and Recipients at Baseline eTable 7. Definitions of Abnormal Outcomes eReferences. [file jamanetwopen-e2030415-s003.pdf]

Supplemental Online Content

Leong KSW, Jayasinghe TN, Wilson BC, et al. Effects of fecal microbiome transfer in adolescents with obesity: the Gut Bugs randomized controlled trial. *JAMA Netw Open*. 2020;3(12):e2030415. doi:10.1001/jamanetworkopen.2020.30415

eTable 1. Android-to-Gynoid-Fat (A/G) Ratio at 6, 12, and 26 Weeks Postintervention Among Female and Male Adolescents

eTable 2. Secondary Outcomes at 6, 12, and 26 Weeks Postintervention

eTable 3. BMI SDS and Glucose Metabolism of Participants with Metabolic Syndrome at Baseline

eTable 4. Changes in Prevalence of Metabolic Syndrome Among Participants with Metabolic Syndrome at Baseline

eTable 5. Variance of Species Taxonomic Profiles (Bray-Curtis Dissimilarity) Explained by Each Covariate in Cross-sectional PERMANOVA

eTable 6. Variance of Species Taxonomic Profiles (Bray-Curtis dissimilarity) Explained by Each Covariate in PERMANOVA for Donors and Recipients at Baseline

eTable 7. Definitions of Abnormal Outcomes

eReferences.

This supplemental material has been provided by the authors to give readers additional information about their work.

eTable 1. Android-to-Gynoid-Fat (A/G) Ratio at 6, 12, and 26 Weeks Postintervention Among Female and Males Adolescents

			**FMT**		**Placebo**			
		**Visit (weeks)**	**N**	**mean ± SD**	**N**	**mean ± SD**	**aMD (95% CI)**	**p-value**
**Females**	**A/G ratio**	**0**	25	1·027 ± 0·088	26	1·032 ± 0·057		
		**6**	24	1·013 ± 0·085	26	1·047 ± 0·074	-0·029 (-0·053, -0·005)	0·018
		**12**	23	1·015 ± 0·084	23	1·054 ± 0·064	-0·028 (-0·054, -0·003)	0·027
		**26**	22	1·008 ± 0·084	24	1·050 ± 0·059	-0·030 (-0·055, -0·006)	0·017
**Males**	**A/G ratio**	**0**	17	1·189 ± 0·078	19	1·131 ± 0·082		
		**6**	15	1·191 ± 0·103	18	1·146 ± 0·106	-0·015 (-0·051, 0·021)	0·41
		**12**	15	1·188 ± 0·078	18	1·148 ± 0·110	-0·023 (-0·060, 0·013)	0·20
		**26**	15	1·173 ± 0·094	17	1·142 ± 0·095	-0·035 (-0·072, 0·003)	0·071

Adjusted mean differences (aMD) and 95% confidence intervals (CI).
FMT, fecal microbiome transfer; SD, standard deviation.
P-values <0.05 were deemed statistically significant.

© 2020 Leong KSW et al. *JAMA Network Open.*

eTable 2. Secondary Outcomes at 6, 12, and 26 Weeks Postintervention

			FMT		Placebo			
		**Visit (weeks)**	**N**	**mean ± SD**	**N**	**mean ± SD**	**aMD (95% CI)**	**p-value**
**24hAMBP**	**Awake SBP (mmHg)**	**0**	42	118·5 ± 8·7	45	118·4 ± 10·0		
		**6**	40	119·4 ± 9·6	43	116·4 ± 8·6	3·0 (0·6, 5·5)	0·015
	**Awake DBP (mmHg)**	**0**	42	69·3 ± 5·0	45	69·8 ± 5·8		
		**6**	40	69·1 ± 6·3	43	68·4 ± 5·9	1·1 (-1·0, 3·2)	0·30
	**Asleep SBP (mmHg)**	**0**	42	107·0 ± 10·9	45	105·4 ± 8·2		
		**6**	40	107·5 ± 10·9	43	105·2 ± 9·3	1·2 (-1·7, 4·2)	0·40
	**Asleep DBP (mmHg)**	**0**	42	57·0 ± 6·1	45	57·4 ± 6·4		
		**6**	40	58·8 ± 7·4	43	57·7 ± 7·1	1·4 (-1·1, 3·8)	0·27
	**Systolic dip (%)**	**0**	42	9·8 ± 6·9	45	10·7 ± 7·1		
		**6**	40	9·9 ± 7·6	43	9·6 ± 6·1	0·7 (-1·9, 3·3)	0·60
	**Diastolic dip (%)**	**0**	42	17·7 ± 8·1	45	17·7 ± 9·3		
		**6**	40	14·7 ± 9·9	43	15·6 ± 9·1	-0·9 (-4·6, 2·8)	0·63
**Lipid profile**	**Total cholesterol (mmol/L)**	**0**	42	4·33 ± 0·97	45	4·41 ± 0·84		
		**6**	39	4·44 ± 0·87	43	4·42 ± 0·86	0·03 (-0·34, 0·40)	0·87
		**12**	37	4·08 ± 0·86	40	4·29 ± 0·82	-0·16 (-0·54, 0·22)	0·41
		**26**	36	4·21 ± 0·84	40	4·23 ± 0·83	0·05 (-0·33, 0·43)	0·80
	**LDL (mmol/L)**	**0**	42	2·61 ± 0·84	45	2·73 ± 0·68		
		**6**	39	2·73 ± 0·77	43	2·74 ± 0·72	0·01 (-0·31, 0·34)	0·94
		**12**	37	2·48 ± 0·77	40	2·77 ± 0·67	-0·23 (-0·56, 0·10)	0·16
		**26**	36	2·62 ± 0·79	40	2·74 ± 0·70	-0·04 (-0·37, 0·29)	0·81
	**HDL (mmol/L)**	**0**	42	1·20 ± 0·27	45	1·23 ± 0·32		
		**6**	39	1·26 ± 0·30	43	1·22 ± 0·25	0·04 (-0·05, 0·14)	0·39
		**12**	37	1·19 ± 0·25	40	1·19 ± 0·25	-0·01 (-0·11, 0·09)	0·88
		**26**	36	1·24 ± 0·26	40	1·17 ± 0·24	0·06 (-0·03, 0·16)	0·20
	**TG (mmol/L)**	**0**	42	1·53 ± 0·80	45	1·23 ± 0·73		
		**6**	39	1·39 ± 0·77	43	1·33 ± 0·77	-0·10 (-0·39, 0·19)	0·48
		**12**	37	1·33 ± 0·82	40	1·23 ± 0·65	0·00 (-0·29, 0·30)	0·98
		**26**	36	1·37 ± 0·90	40	1·31 ± 0·66	-0·04 (-0·34, 0·25)	0·78
**Liver function**	**ALT (U/L)**	**0**	42	26·6 ± 14·6	45	22·1 ± 11·6		
		**6**	39	28·8 ± 20·0	43	26·4 ± 15·6	1·1 (-6·0, 8·3)	0·76
		**12**	37	28·9 ± 20·6	40	25·3 ± 15·5	2·6 (-4·8, 9·9)	0·49
		**26**	36	32·1 ± 20·3	40	25·9 ± 15·8	4·2 (-3·2, 11·7)	0·26
	**AST (U/L)**	**0**	42	29·0 ± 9·1	45	26·3 ± 10·2		
		**6**	39	28·3 ± 11·7	43	27·9 ± 7·0	0·4 (-3·7, 4·5)	0·86
		**12**	37	28·0 ± 11·9	40	24·8 ± 7·7	2·9 (-1·3, 7·2)	0·17
		**26**	36	28·1 ± 11·6	40	27·4 ± 9·5	-0·2 (-4·5, 4·1)	0·92
	**GGT (U/L)**	**0**	42	33·6 ± 21·7	45	28·0 ± 16·3		
		**6**	39	34·7 ± 26·9	43	25·3 ± 12·0	6·6 (-0·1, 13·2)	0·052
		**12**	37	32·8 ± 27·2	40	24·9 ± 10·7	5·1 (-1·7, 11·9)	0·14
		**26**	36	32·9 ± 24·4	40	24·9 ± 12·8	5·0 (-1·9,11·9)	0·15
**Inflammatory markers**	**Uric acid (umol/L)**	**0**	42	418 ± 88	45	394 ± 73		

© 2020 Leong KSW et al. *JAMA Network Open.*

			FMT		Placebo			
		**Visit (weeks)**	**N**	**mean ± SD**	**N**	**mean ± SD**	**aMD (95% CI)**	**p-value**
		**6**	39	396 ± 90	43	404 ± 84	-8 (-40, 25)	0·64
		**12**	37	391 ± 107	40	404 ± 76	-13 (-46, 20)	0·44
		**26**	36	398 ± 85	40	410 ± 85	-10 (-44, 23)	0·54
	**hsCRP (mg/L)**	**0**	42	3·32 ± 3·50	45	3·30 ± 3·69		
		**6**	39	3·54 ± 3·54	43	3·64 ± 4·37	-0·10 (-1·75, 1·56)	0·91
		**12**	37	3·02 ± 2·93	40	3·45 ± 3·47	-0·68 (-2·37, 1·02)	0·43
		**26**	36	3·52 ± 3·48	40	4·12 ± 4·57	-0·81 (-2·53, 0·92)	0·36

Adjusted mean differences (aMD) and 95% confidence intervals (CI).
24hAMBP, 24-hour ambulatory blood pressure monitoring; ALT, alanine transaminase; AST, aspartate transaminase; DBP, diastolic blood pressure; GGT, gamma-glutamyl transferase; FMT, fecal microbiome transfer; HDL; high-density lipoprotein cholesterol; hsCRP, high-sensitivity C-reactive protein; LDL, low-density lipoprotein cholesterol; SBP, systolic blood pressure; SD, standard deviation; TG, triglycerides.
P-values <0.05 were deemed statistically significant.

© 2020 Leong KSW et al. *JAMA Network Open.*

eTable 3: BMI SDS and Glucose Metabolism of Participants With Metabolic Syndrome at Baseline

			FMT		Placebo			
		**Visit (weeks)**	**N**	**mean (95% CI)**	**N**	**mean (95% CI)**	**aMD (95% CI)**	**p-value**
**Anthropometry**	**BMI SDS**	**0**	18	4.01 (3.63, 4.38)	13	3.09 (2.66, 3.53)	0.91 (0.34, 1.49)	<0.0001
		**6**	17	3·72 (3·63, 3·80)	13	3·70 (3·60, 3·81)	0·01 (-0·13, 0·15)	0·88
		**12**	17	3·70 (3·61, 3·79)	12	3·71 (3·60, 3·82)	-0·01 (-0·15, 0·14)	0·93
		**26**	17	3·74 (3·65, 3·82)	12	3·71 (3·60, 3·82)	-0·01 (-0·15, 0·14)	0·93
**Glucose metabolism**	**Fasting insulin (µU/ml)**	**0**	18	35·5 (27·2, 46·2)	13	28·8 (21·2, 39·2)	1·23 (0·82, 1·84)	0·30
		**6**	16	27·7 (22·3, 34·4)	13	39·0 (30·5, 49·9)	0·71 (0·51, 0·99)	0·042
		**12**	13	36·1 (28·6, 45·5)	12	33·4 (26·0, 43·0)	1·08 (0·77, 1·52)	0·66
		**26**	15	31·2 (25·0, 39·0)	12	35·4 (27·5, 45·6)	0·88 (0·63, 1·23)	0·45
	**Fasting glucose (mmol/L)**	**0**	18	5·42 (5·21, 5·64)	13	5·58 (5·33, 5·83)	-0·16 (-0·49, 0·17)	0·33
		**6**	16	5·15 (4·92, 5·39)	13	5·53 (5·28, 5·79)	-0·38 (-0·73, -0·03)	0·033
		**12**	13	5·22 (4·96, 5·47)	12	5·51 (5·24, 5·78)	-0·29 (-0·66, 0·08)	0·12
		**26**	15	5·44 (5·20, 5·68)	12	5·55 (5·29, 5·82)	-0·12 (-0·48, 0·24)	0·52
	**HOMA-IR**	**0**	18	8·53 (6.49, 11.22)	13	7·12 (5.18, 9.79)	1·20 (0·79, 1·82)	0·38
		**6**	16	6·29 (5.00, 7.92)	13	9·60 (7·41, 12·43)	0·66 (0·46, 0·93)	0·018
		**12**	13	8·27 (6·47, 10·58)	12	8·13 (6·23, 10·61)	1·02 (0·71, 1·46)	0·93
		**26**	15	7·49 (5·92, 9·48)	12	8·69 (6·66, 11·34)	0·86 (0·60, 1·23)	0·41
	**Matsuda index**	**0**	18	1·14 (0.87, 1.51)	13	1·25 (0.90, 1.72)	0·92 (0·60, 1·40)	0·68
		**6**	16	1·45 (1.21, 1.73)	13	1·11 (0·91, 1·35)	1·30 (1·00, 1·71)	0·053
		**12**	13	1·13 (0·93, 1·38)	12	1·31 (1·07, 1·62)	0·86 (0·65, 1·15)	0·30
		**26**	15	1·14 (0·95, 1·38)	12	1·16 (0·94, 1·43)	0·98 (0·74, 1·30)	0·90

Group data for BMI SDS and fasting glucose are adjusted means and 95% confidence intervals (CI), adjusted for sex; for the same parameters, the adjusted mean differences (aMD) are means and 95% CI adjusted for sex and the baseline value of the outcome (except for the aMD for baseline).

Group data for fasting insulin, HOMA-IR, and Matsuda index are the geometric means and 95% CI (back-transformed from logged data), adjusted for sex; the aMD for the same parameters are also geometric means and 95% CI, adjusted for sex and the baseline value of the outcome.

BMI SDS, body mass index standard deviation score; FMT, fecal microbiome transfer; HOMA-IR, homeostatic model assessment of insulin resistance.

P-values <0.05 were deemed statistically significant.

© 2020 Leong KSW et al. *JAMA Network Open.*

eTable 4: Changes in Prevalence of Metabolic Syndrome Among Participants with Metabolic Syndrome at Baseline

		FMT	Placebo		
	**Visit (weeks)**	**n (%)**	**n (%)**	**aOR (95% CI)**	**p-value**
**Metabolic syndrome ♦**	**0**	18 (100%)	13 (100%)		
	**6**	13 (81%)	9 (69%)	2.00 (0·32, 12.69)	0·45
	**12**	5 (38%)	7 (58%)	0.42 (0·07, 2·50)	0·33
	**26**	4 (27%)	10 (83%)	0·06 (0·01, 0.45)	0·0074

Adjusted odds ratios (aOR) and 95% confidence intervals (CI).
FMT, fecal microbiome transfer
♦ Metabolic syndrome outcome is defined in eTable 6.
P-values <0.05 were deemed statistically significant.

© 2020 Leong KSW et al. *JAMA Network Open.*

eTable 5. Variance of Species Taxonomic Profiles (Bray-Curtis Dissimilarity) Explained by Each Covariate in Cross-Sectional PERMANOVA

	Baseline (n = 84)			Week 6 (n = 82)			Week 12 (n = 77)			Week 26 (n = 73)		
**Covariates**	**R^2**	**p-value**	**q-value**	**R^2**	**p-value**	**q-value**	**R^2**	**p-value**	**q-value**	**R^2**	**p-value**	**q-value**
**Sequence batch**	4·71%	0·057	0·15	3·21%	0·086	0·18	n/a	n/a	n/a	n/a	n/a	n/a
**Sex**	1·29%	0·31	0·40	3·70%	0·001	**0·013**	1·32%	0·37	0·46	1·71%	0·18	0·28
**Age**	1·53%	0·16	0·28	0·55%	0·96	0·96	1·69%	0·17	0·28	3·30%	0·008	**0·055**
**Ethnicity**	6·45%	0·032	0·14	6·23%	0·050	0·15	6·86%	0·060	0·15	5·98%	0·22	0·31
**Antibiotics**	NA	NA	NA	1·49%	0·18	0·28	1·03%	0·64	0·71	1·22%	0·49	0·57
**Treatment group**	0·59%	0·95	0·96	2·73%	0·006	0·055	2·94%	0·011	0·060	2·23%	0·063	0·15

Table contain R^2 (the proportion of variance explained), nominal p-value based on 10,000 permutation tests, and FDR-adjusted p-values (q-values) after multiple comparisons.
q-values <0.1 are statistically significant.
n/a, not applicable.

© 2020 Leong KSW et al. *JAMA Network Open.*

eTable 6. Variance of Species Taxonomic Profiles (Bray-Curtis Dissimilarity) Explained by Each Covariate in PERMANOVA for Donors and Recipients at Baseline

Covariates	R^2	p-value	q-value
**Sex**	0.91%	0·566	0·566
**Ethnicity**	5.55%	0·045	**0·068**
**Participant type***	1.80%	0·042	**0·068**

Table contain R^2 (the proportion of variance explained), nominal p-value based on 10,000 permutation tests, and FDR-adjusted p-values (q-values) after multiple comparisons.
Significant findings are highlighted in bold (q value <0·1).

*Participant type compared the microbiomes of 9 donors with 87 participants at baseline.

© 2020 Leong KSW et al. *JAMA Network Open.*

eTable 7. Definitions of Abnormal Outcomes

OUTCOMES	THRESHOLDS FOR ABNORMAL OUTCOMES	REFERENCE
**Abnormal glycaemia**	Fasting glucose ≥5.6 mmol/L or 2-hour glucose ≥7.8 mmol/L or HbA1c ≥39 mmol/mol	American Diabetes Association 2018[1]
**Elevated clinic BP**	<16 years: SBP and/or DBP ≥90th for age and sex	Lurbe et al. 2016[2]
	≥16 years: SBP ≥130 and/or DBP ≥85 mmHg	
**Low HDL**	<16 years: <1.03 mmol/L	Zimmet et al. 2007[3]
	≥16 years: males <1.03 mmol/L; females <1.29 mmol/L	
**High LDL**	>2.6 mmol/L	NCEP 2001[4]
**High triglycerides**	≥1.7 mmol/L	Zimmet et al. 2007[3]
**High total cholesterol**	>5.2 mmol/L	European Atherosclerosis Society 1987[5]
**Dyslipidaemia**	Low HDL or high LDL or high triglycerides or high total cholesterol	
**Elevated ALT**	Males >41 U/L; females >33 U/L	Klein et al. 1994[6]
**Elevated AST**	Males >40 U/L, females >32 U/L	Thefeld et al. 1974[7]
**Elevated GGT**	Males ≥60 U/L, females ≥40 U/L	Thomas et al. 2005[8]
**Abnormal liver function**	Elevated ALT or elevated AST or elevated GGT	
**Metabolic syndrome**	≥10 but <16 years:<br>Waist circumference ≥90th percentile (or adult cut-off if the latter is lower); AND any 2 of the following 4 criteria:<br>1. triglycerides ≥1.7 mmol/L<br>2. HDL <1.03 mmol/L<br>3. SBP ≥130 and/or DBP ≥85 mmHg<br>4. Fasting glucose ≥5.6 mmol/L and/or previously diagnosed type 2 diabetes	Zimmet et al. 2007[3]
	≥16 years:<br>Waist circumference ≥94 cm for males and ≥80 cm for females; AND any 2 of the following 4 criteria:<br>1. triglycerides ≥1.7mmol/L<br>2. HDL <1.03 mmol/L in males and <1.29 mmol/L in females; or specific treatment for these lipid abnormalities<br>3. SBP ≥130 mmHg and/or DBP ≥85 mmHg, or treatment for previously diagnosed hypertension<br>4. Fasting glucose ≥5.6 mmol/L and/or previously diagnosed type 2 diabetes	

24hABPM, 24-hour ambulatory blood pressure monitoring; ALT, alanine transaminase; AST, aspartate transaminase; BP, blood pressure; DBP, diastolic blood pressure; GGT, gamma-glutamyl transferase; hsCRP, high-sensitivity C-reactive protein; HbA1c, haemoglobin A1c; HDL; high-density lipoprotein cholesterol; LDL, low-density lipoprotein cholesterol; SBP, systolic blood pressure.

eReferences

1. American Diabetes Association. Classification and Diagnosis of Diabetes: Standards of Medical Care in Diabetes 2018. *Diabetes Care.* 2018;41(Supplement 1):S13-S27.
2. Lurbe E, Agabiti-Rosei E, Cruickshank JK, et al. 2016 European Society of Hypertension guidelines for the management of high blood pressure in children and adolescents. *J Hypertens.* 2016;34(10):1887-1920.
3. Zimmet P, Alberti KGM, Kaufman F, et al. The metabolic syndrome in children and adolescents–an IDF consensus report. *Pediatr Diabetes.* 2007;8(5):299-306.
4. NCEP Expert Panel. Executive Summary of The Third Report of The National Cholesterol Education Program (NCEP) Expert Panel on Detection, Evaluation, And Treatment of High Blood Cholesterol In Adults (Adult Treatment Panel III). *JAMA.* 2001;285(19):2486-2497.
5. European Atherosclerosis Society. Strategies for the prevention of coronary heart disease: a policy statement of the European Atherosclerosis Society. *Eur Heart J.* 1987;8(1):77-88.
6. Klein G, Lehmann P, Michel E, Regenauer H. [Comparison of the IFCC methods for ALT, AST and GGT at 37°C with the established standard methods at 25°C and 37°C]. *Lab Med.* 1994;18:403-404.
7. Thefeld W, Hoffmeister H, Busch E-W, Koller P, Vollmar J. [Reference values for the determinations of the transaminases GOT and GPT as well as serum alkaline phosphatase using optimized standard methods]. *Dtsch Med Wochenschr.* 1974;99(8):343-351.
8. Thomas L, Müller M, Schumann G, et al. Consensus of DGKL and VDGH for interim reference intervals on enzymes in serum *J Lab Med.* 2005;29(5):301-308.

© 2020 Leong KSW et al. *JAMA Network Open.*
