# Supplementary material for: Effects of Fecal Microbiome Transfer in Adolescents With Obesity: The Gut Bugs Randomized Controlled Trial
Source: JAMA Netw Open. 2020 Dec 21;3(12):e2030415. doi: 10.1001/jamanetworkopen.2020.30415 (PMC7753902; doi:10.1001/jamanetworkopen.2020.30415)
Supplement: Supplement 4. — Data Sharing Statement [file jamanetwopen-e2030415-s004.pdf]

Data Sharing Statement

Leong KSW, Jayasinghe TN, Wilson BC, et al. Effects of fecal microbiome transfer in adolescents with obesity: the Gut Bugs randomized controlled trial. *JAMA Netw Open*. 2020;3(12):e2030415. doi:10.1001/jamanetworkopen.2020.30415

Data available: Yes
Data types: Deidentified participant data, Other (please specify)
Additional Information: De-identified metagenomic sequencing data
How to access data: De-identified metagenomic sequencing data are available in the NCBI Sequence Read Archive under BioProject. The BioProject accession is: PRJNA637785. The anonymized clinical data could be made available to other investigators upon bona fide request, and following all the necessary approvals (including ethics) of the detailed study proposal and statistical analyses plan. Any queries should be directed to Prof Wayne Cutfield (w.cutfield@auckland.ac.nz) or A/P Justin O'Sullivan (justin.osullivan@auckland.ac.nz).
When available: With publication

Supporting Documents
Document types: None

Additional Information
Who can access the data: Researchers whose proposed use of the data fits within the ethical approval and participant agreements.
Types of analyses: Specified purpose
Mechanisms of data availability: After approval of a proposal.
Any additional restrictions: N/A
